# Drought‐induced protein (Di19‐3) plays a role in auxin signaling by interacting with IAA14 in Arabidopsis

**DOI:** 10.1002/pld3.234

**Published:** 2020-06-21

**Authors:** Susmita Maitra Majee, Eshan Sharma, Brinderjit Singh, Jitendra P. Khurana

**Affiliations:** ^1^ Interdisciplinary Centre for Plant Genomics & Department of Plant Molecular Biology University of Delhi South Campus New Delhi India

**Keywords:** abiotic stress, Arabidopsis, Aux/IAA, auxin signaling, Di19, ethylene signaling

## Abstract

The members of early auxin response gene family, *Aux/IAA*, encode negative regulators of auxin signaling but play a central role in auxin‐mediated plant development. Here we report the interaction of an Aux/IAA protein, AtIAA14, with Drought‐induced‐19 (Di19‐3) protein and its possible role in auxin signaling. The *Atdi19‐3* mutant seedlings develop short hypocotyl, both in light and dark, and are compromised in temperature‐induced hypocotyl elongation. The mutant plants accumulate more IAA and also show altered expression of *NIT2*, *ILL5*, and *YUCCA* genes involved in auxin biosynthesis and homeostasis, along with many auxin responsive genes like *AUX1* and *MYB77*. *Atdi19‐3* seedlings show enhanced root growth inhibition when grown in the medium supplemented with auxin. Nevertheless, number of lateral roots is low in *Atdi19‐3* seedlings grown on the basal medium. We have shown that AtIAA14 physically interacts with AtDi19‐3 in yeast two‐hybrid (Y2H), bimolecular fluorescence complementation, and in vitro pull‐down assays. However, the auxin‐induced degradation of AtIAA14 in the *Atdi19‐3* seedlings was delayed. By expressing *pIAA14::mIAA14‐GFP* in *Atdi19‐3* mutant background, it became apparent that both Di19‐3 and AtIAA14 work in the same pathway and influence lateral root development in Arabidopsis. Gain‐of‐function *slr‐1/iaa14* (*slr*) mutant, like *Atdi19‐3*, showed tolerance to abiotic stress in seed germination and cotyledon greening assays. The *Atdi19‐3* seedlings showed enhanced sensitivity to ethylene in triple response assay and AgNO_3_, an ethylene inhibitor, caused profuse lateral root formation in the mutant seedlings. These observations suggest that AtDi19‐3 interacting with AtIAA14, in all probability, serves as a positive regulator of auxin signaling and also plays a role in some ethylene‐mediated responses in Arabidopsis.

**Significance Statement:**

This study has demonstrated interaction of auxin responsive Aux/IAA with Drought‐induced 19 (Di19) protein and its possible implication in abiotic stress response.

## 1 INTRODUCTION

Hormones play a major role in coordinating various developmental processes in plants as well as in adaptation of plants to both abiotic and biotic stresses. There is a complex network of signaling components that act as a link between the signaling pathways of various hormones, thus relaying the signal to the nucleus, eliciting changes in gene expression, and leading eventually to more overt developmental responses. Auxin is one of the major phytohormones that has been studied extensively and is involved in many developmental responses, including embryonic and post‐embryonic development and tropic movements in plants. Relative content of auxin in a particular tissue and at a specific stage of development is decisive in defining the fate of cells in that tissue. However, high levels of indole‐3‐acetic acid (IAA) at the cellular level induce various growth abnormalities like reduction in shoot growth, epinasty, reduction in leaf area, and root proliferation (Grossmann & Scheltrup, 1998); this also forms the very basis of herbicidal action of auxins. Moreover, in coordination with other hormones like ethylene, gibberellin, abscisic acid, and cytokinin, auxin plays an essential role in many fundamental processes in plants (Chandler, 2009; Munné‐Bosch & Müller, 2013), conferring phenotypic plasticity. Synergy between auxin and ethylene can be explained by positive regulation of auxin synthesis by ethylene (Stepanova, Hoyt, Hamilton, & Alonso, 2005; Stepanova, Yun, Likhacheva, & Alonso, 2007) along with involvement of ethylene in polar auxin transport (Lewis, Negi, Sukumar, & Muday, 2011; Prayitno, Rolfe, & Mathesius, 2006; Strader, Chen, & Bartel, 2010). Moreover, auxin regulated *AUX1* has been implicated as a central player in ethylene‐induced inhibition of lateral root (LR) formation (Lewis et al., 2011; Negi, Ivanchenko, & Muday, 2008). Recently, the role of SOR1, an E3 ubiquitin ligase, in ethylene–auxin‐mediated inhibition of root growth in rice seedlings has been demonstrated (Chen et al., 2018). Auxin has been shown to induce ethylene production by upregulating ACC synthase expression or by redirecting the available pool of ACC towards ethylene synthesis (Stepanova et al., 2007; Wei, Zheng, & Hall, 2000). Auxin also stimulates the expression of NCED encoding gene leading to ABA accumulation (Grossmann & Scheltrup, 1998; Hansen & Grossmann, 2000; Kraft, Kuglitsch, Kwiatkowski, Frank, & Grossmann, 2007). In fact, in earlier studies from our laboratory (Borah et al., 2017; Jain & Khurana, 2009), many of the auxin responsive genes in rice were found to be expressed differentially under abiotic stress conditions like drought, salinity, cold, and heat stress. Many other recent studies have also provided evidences for the involvement of auxin in abiotic stress response pathways (Jung, Lee, Choi, & Kim, 2015; Shani et al., 2017; Sharma, Sharma, Borah, Jain, & Khurana, 2015; Uga et al., 2013).

The changes in the endogenous level of auxin can cause both repression and enhanced expression of auxin responsive genes. Among the genes that are upregulated, members of *Aux/IAA*, *GH3*, and *SAUR* gene families have been the subject of innumerable studies (Hagen & Guilfoyle, 2002; Jain, Kaur, Garg, et al., 2006; Jain, Kaur, Tyagi, & Khurana, 2006; Jain & Khurana, 2009; Jain, Tyagi, & Khurana, 2006; Paponov et al., 2008). Auxin‐induced changes in gene expression are mediated by two well‐studied gene families encoding Aux/IAAs and auxin response factors (ARFs). The Aux/IAA family represents auxin‐induced short lived proteins that act as negative regulators of auxin signaling (Gray, Kepinski, Rouse, Leyser, & Estelle, 2001; Jain, Kaur, Garg, et al., 2006; Ramos, Zenser, Leyser, & Callis, 2001; Santos Maraschin, Memelink, & Offringa, 2009). The expression of most of the *Aux/IAA* genes is enhanced upon auxin treatment and at the same time it signals the activation of proteasome pathway. Auxin finds a binding pocket in its receptor TIR1/AFB, acting as a “molecular glue,” thus providing an extended interface for the protein–protein interaction between Aux/IAA and TIR1/AFB (Dharmasiri, Dharmasiri, & Estelle, 2005; Kepinski & Leyser, 2005; Tan et al., 2007). Auxin enhances TIR1/AFB protein binding to Aux/IAA for its degradation that hitherto has sequestered the ARFs, thus leaving the ARFs free to bind *AuxREs* in the upstream promoter region of auxin responsive genes and regulate their expression. Considering the complexity of auxin signaling and its participation in a wide diversity of physiological processes and developmental events, it is not surprising that many more components may be involved in auxin signaling pathways. Recent studies have led to the identification of components that serve as chaperones and chromatin modifiers, affecting auxin signaling (Lavy & Estelle, 2016). A mechanism involving the Mediator complex, which becomes functional only on degradation of IAA14 at higher levels of auxin, due to dissociation from TPL and CKM, thus allowing ARF7‐ and ARF19‐mediated gene expression to commence, has been deciphered recently (Ito et al., 2016).

In an effort to identify Aux/IAA interacting proteins and define their cellular functions, in the present study, we initially identified an interacting partner, Di19, for one of the Aux/IAA proteins (OsIAA13) in *Oryza sativa* through Y2H library screening and subsequently confirmed it by in vitro pull‐down and bimolecular fluorescence complementation (BiFC) assays. Based on the lead available from rice work, we could identify the rice homologs of Di19 and Aux/IAA in *Arabidopsis thaliana* and also demonstrated their interaction. Di19 family proteins represent a class of Cys2/His2 zinc finger transcription factors that are induced upon drought stress (Milla, Townsend, Chang, & Cushman, 2006). In Arabidopsis as well as rice, there are seven hydrophilic members comprising the Di19 family. The transcript levels of *Di19* family genes are enhanced upon dehydration and high salinity stress (Milla et al., 2006; Wang et al., 2014). There are also reports showing association of AtDi19‐1 with pathogenesis response (Liu et al., 2013). To pursue the work on Arabidopsis, we searched for an Arabidopsis mutant of the closest homolog of OsDi19‐5 in Arabidopsis; AtDi19‐3 emerged as the closest match to OsDi19‐5. Subsequent analysis of overexpression, complementation and knock down mutant seedlings of *AtDi19‐3* clearly revealed its involvement in various developmental events, like hypocotyl growth, LR growth, and development. Furthermore, the roles of AtDi19‐3 in auxin‐ and ethylene‐mediated responses have also been analyzed. Overall, our work provides evidence that Di19‐3 is involved in hormonal interplay for regulating plant development besides the role it might play in stress response pathways.

## 2 RESULTS

### 2.1 OsIAA13 interacts with OsDi19‐5 and AtIAA14 interacts with AtDi19‐3

**FIGURE 1:**
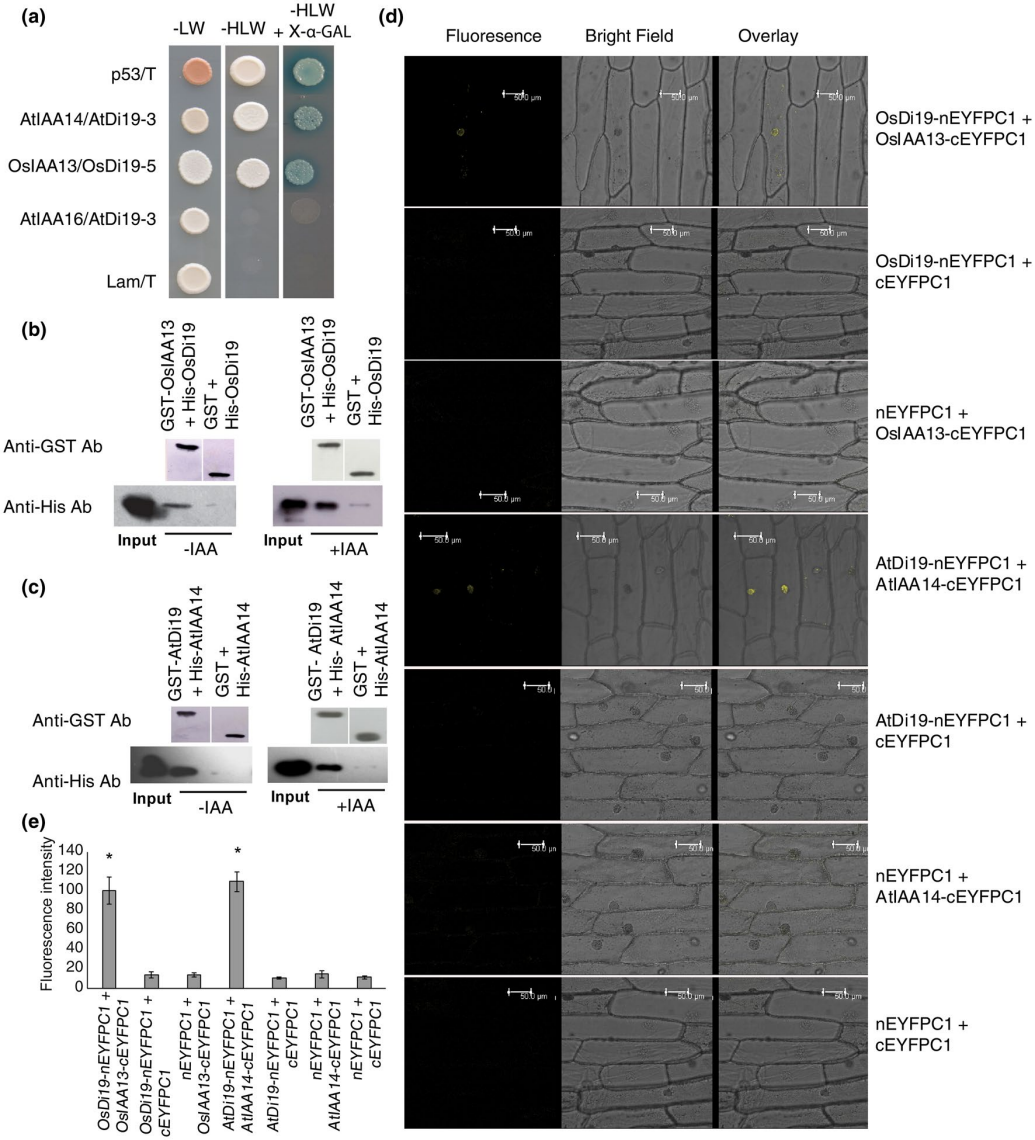
Physical interaction between Aux/IAA and Di19. (a) Yeast two‐hybrid assay showing interaction of AtIAA14 and OsIAA13 with AtDi19‐3 and OsDi19‐5, respectively; AtIAA16 was used as a negative control. The assay was performed in the presence of *SD*/‐LW as well as *SD*/‐HLW for *HIS3* expression and *MEL1* expression. p53/T interaction and Lam/T interaction serve as positive and negative control, respectively. (b) In vitro pull‐down assay of OsDi19‐5 with OsIAA13 immobilized on GSH Sepharose. Recombinant 6xHis‐OsDi19‐5 was passed through GST‐OsIAA13 and GST bound Sepharose beads in the absence and presence of 5µM IAA and the presence of 50 µM MG132 (a 26S proteasome inhibitor), followed by anti‐His antibody immunodetection; (c) 6xHis‐AtIAA14 was immunoblotted after passing through GST‐AtDi19‐3 and GST bound Sepharose beads. Immunodetection with anti‐His antibody revealed that AtIAA14 interacts with AtDi19‐3; (d) Bimolecular fluorescence complementation analysis of Aux/IAA and Di19. Confocal images of onion epidermal cells bombarded with i) OsIAA13/cYFPC1 and OsDi19‐5/nYFPC1; ii) cYFPC1 and OsDi19‐5/nYFPC1; iii) nYFPC1 and OsIAA13/cYFPC1; iv) AtIAA14/cYFPC1 and AtDi19‐3/nYFPC1; v) cYFPC1 and AtDi19‐3/nYFPC1; vi) nYFPC1 and AtIAA14/cYFPC1; vii) cYFPC1 and nYFPC1; bar is 50µm; (e) Fluorescence intensity quantified using Leica LAS AF Lite software 4.0 plotted with mean values ± SE. One‐way ANOVA showed significant statistical difference; *represents *p* < .05 (Student's *t* test)

In pursuit of identifying different components in Aux/IAA‐mediated auxin signaling, we performed library screening in yeast using one of the rice Aux/IAAs as bait. With prior knowledge that *OsIAA13* (earlier designated as *OsIAA1*; Thakur, Tyagi, & Khurana, 2001) has high expression in coleoptiles of 3‐day‐old etiolated seedlings of rice and it is induced by auxin (Thakur, Jain, Tyagi, & Khurana, 2005; Thakur et al., 2001), we generated cDNA library from 3‐day‐old etiolated rice seedlings for yeast two‐hybrid (Y2H) screening with OsIAA13 as bait. In addition to OsDi19‐5, we found few ARFs, a dnaJ, and a CaMK to be interacting with OsIAA13; in this study, we kept our focus on OsDi19‐5. The interaction between OsIAA13 and OsDi19‐5 was further analyzed by Y2H, in vitro pull‐down, and BiFC assays (Figure 1a–e). For in vitro pull‐down assay, Sepharose 4B pre‐cleared 6X‐His tagged OsDi19‐5 was passed through GSH‐Sepharose 4B beads bound GST‐OsIAA13 or GST. Immuno‐detection with anti‐His antibody revealed that OsDi19‐5 could be affinity purified by GST‐OsIAA13 protein; GST alone did not show any affinity to OsDi19‐5 (Figure 1b). To confirm the interaction under in vivo conditions, BiFC assay was performed using split YFP. Full‐length sequence of *OsIAA13* fused to N‐terminal of YFP and *OsDi19‐5* fused with C‐terminal of YFP were co‐bombarded into onion epidermal cells and viewed under confocal microscope after incubation for the appropriate duration. The detection of YFP fluorescence in the nucleus (Figure 1d,e; Figure S8) confirmed the physical interaction between these two proteins.

In an earlier study, the Arabidopsis Di19 family genes coding for Cys2/His2 zinc finger class of transcription factors, have been shown to be upregulated upon exposure to dehydration and high salinity stress (Milla et al., 2006). Since genetic resources are readily available for Arabidopsis, we extended this work on Arabidopsis and first identified the homologs of OsIAA13 and OsDi19‐5 in Arabidopsis. Using Matrix Global Alignment tool (MatGAT) (Campanella, Bitincka, & Smalley, 2003), AtDi19‐3 showed the closest match to OsDi19‐5 (Table S1). Likewise, phylogenetic analysis revealed that OsIAA13 shares the clade with AtIAA14 (Figure S3).

The physical interaction between AtDi19‐3 with AtIAA14 was confirmed in yeast (Figure 1a); AtIAA16, another Aux/IAA protein belonging to the same clade, however, did not show any interaction with AtDi19‐3, and thus served as a negative control. Among the seven members of AtDi19 family, AtIAA14 was found to interact predominantly with AtDi19‐3 (Figure S1c). Affinity purification of 6X‐His‐AtIAA14 using GST‐AtDi19‐3 bound to GSH‐Sepharose and subsequent immunodetection confirmed their interaction by in vitro pull‐down assay (Figure 1c). The presence of YFP fluorescence in the nucleus of onion epidermal cells during BiFC assay further substantiated the interaction between AtIAA14 and AtDi19‐3 (Figure 1d,e; Figure S8). These observations provide sufficient evidence for the interaction between AtIAA14 and AtDi19‐3 in Arabidopsis. Thus, further work was only confined to Arabidopsis; work on rice we intend to pursue later.

### 2.2 Altered hypocotyl elongation in *Atdi19‐3* mutant seedlings grown in light and dark and in response to changes in temperature

To elucidate whether the interaction of AtDi19‐3 with AtIAA14 is physiologically relevant for auxin‐regulated responses, mutant of *AtDi19‐3* (SALK_072390) was obtained from ABRC. Following the standard procedure for SALK T‐DNA insertion lines, homozygous line for *Atdi19‐3* was identified and the insertion site was mapped near the 3' end of the fifth exon of the coding sequence (Figure S2a). The transcript level of At*Di19‐3* was considerably reduced in *Atdi19‐3* homozygous line as revealed by real time‐quantitative PCR (RT‐qPCR) (Figure S2b). In addition, we constitutively expressed *AtDi19‐3* in wild‐type Col‐0, as well as complemented *Atdi19‐3* mutant with *AtDi19‐3* and *OsDi19‐5* genes from Arabidopsis and rice, respectively. The *AtDi19‐3* transcript accumulation in *Atdi19‐3/35S:AtDi19‐3* complimented lines was almost same as in the wild‐type, whereas the overexpression lines, *35S:AtDi19* (L2 and L5), showed significant increase in transcript level compared to the wild‐type plants (Figure S2b).

**FIGURE 2:**
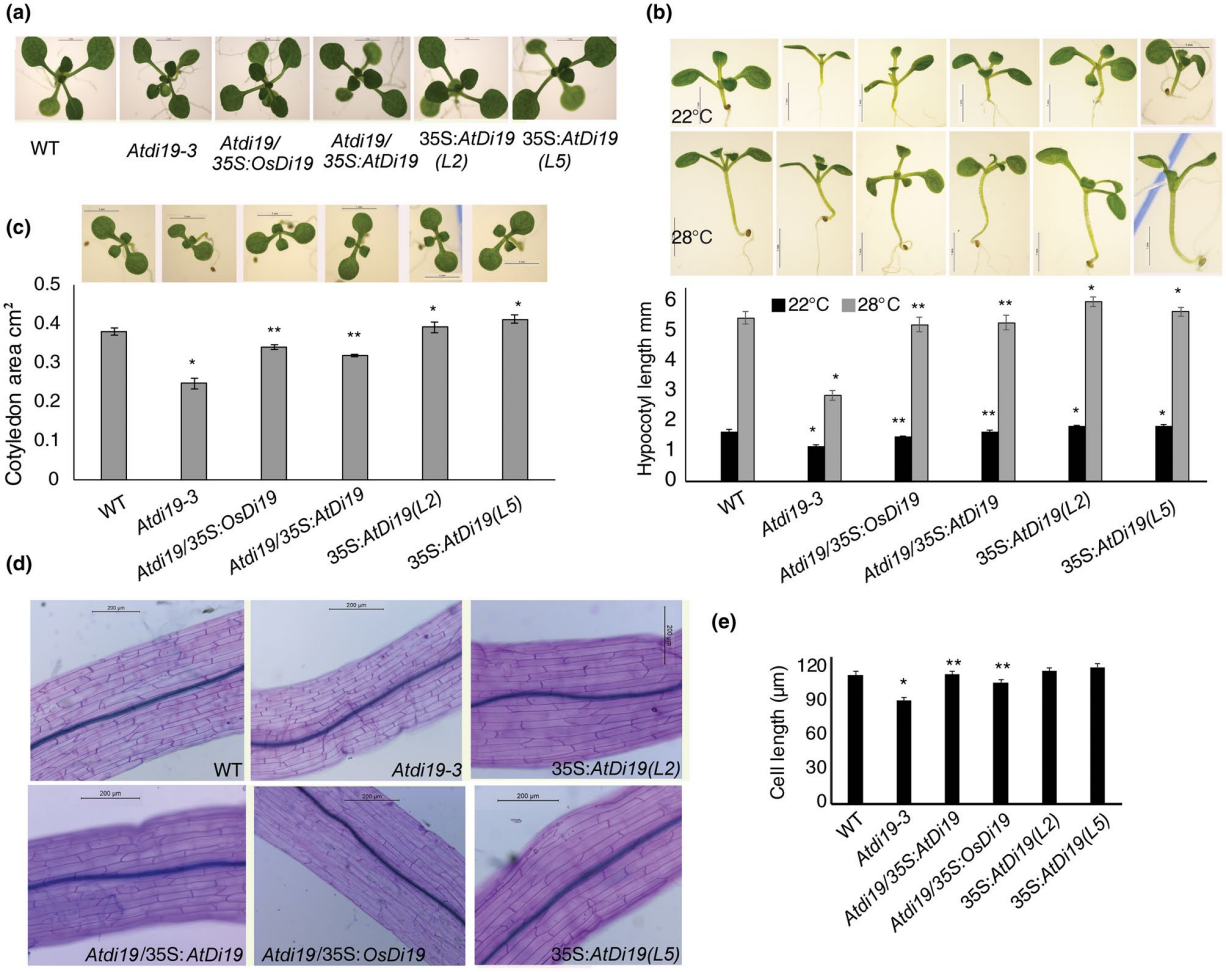
Comparative phenotypic analysis of Arabidopsis *di19‐3* mutant and wild‐type seedlings grown under light conditions. (a) Phenotype of 14‐day‐old seedlings (top view); (b) Phenotype of 7‐day‐old light grown seedlings depicting the effect of temperature (at 22 and 28°C) on hypocotyl elongation growth; (c) cotyledon area. Mean value ± SE of three independent experiments with 20 seedlings in each set was plotted. (d and e) Hypocotyl epidermal cells of 7‐day‐old light grown seedlings. Mean value (±SE) of three different experiments was plotted. One‐way ANOVA was performed followed by Student's *t* test (both at *p* < .05) for estimation of statistical significance (*wild‐type vs. mutant/overexpressing lines; **mutant vs. complementation lines)

Seedlings/plants were grown in white light (50–60 µmol m^−2^ s^−1^) for comparative phenotypic analysis. The rosette size of 14‐day‐old plants of *Atdi19‐3* mutant was smaller in comparison to plants of the wild‐type and the complemented lines; however, in overexpression lines the rosette was somewhat larger in size (Figure 2a). The fully mature *Atdi19‐3* plants were also short in height (Figure S2c). The phenotypic differences were more apparent in young *Atdi19‐3* seedlings grown in white light. The hypocotyl length was shorter than the wild‐type, whereas the complemented lines had hypocotyl length similar to the wild‐type (Figure 2b); however, *35S:Di19* (lines L2 & L5) seedlings showed slight increase in hypocotyl length (Figure 2b). The cotyledon size was also relatively smaller in *Atdi19‐3* seedlings (Figure 2c). The epidermal cell size of *Atdi19‐3* mutant seedling hypocotyls was also smaller than the wild‐type. The cell size of complemented lines as well as overexpressing lines was more or less similar to the wild‐type (Figure 2d,e; Figure S4a).

**FIGURE 3:**
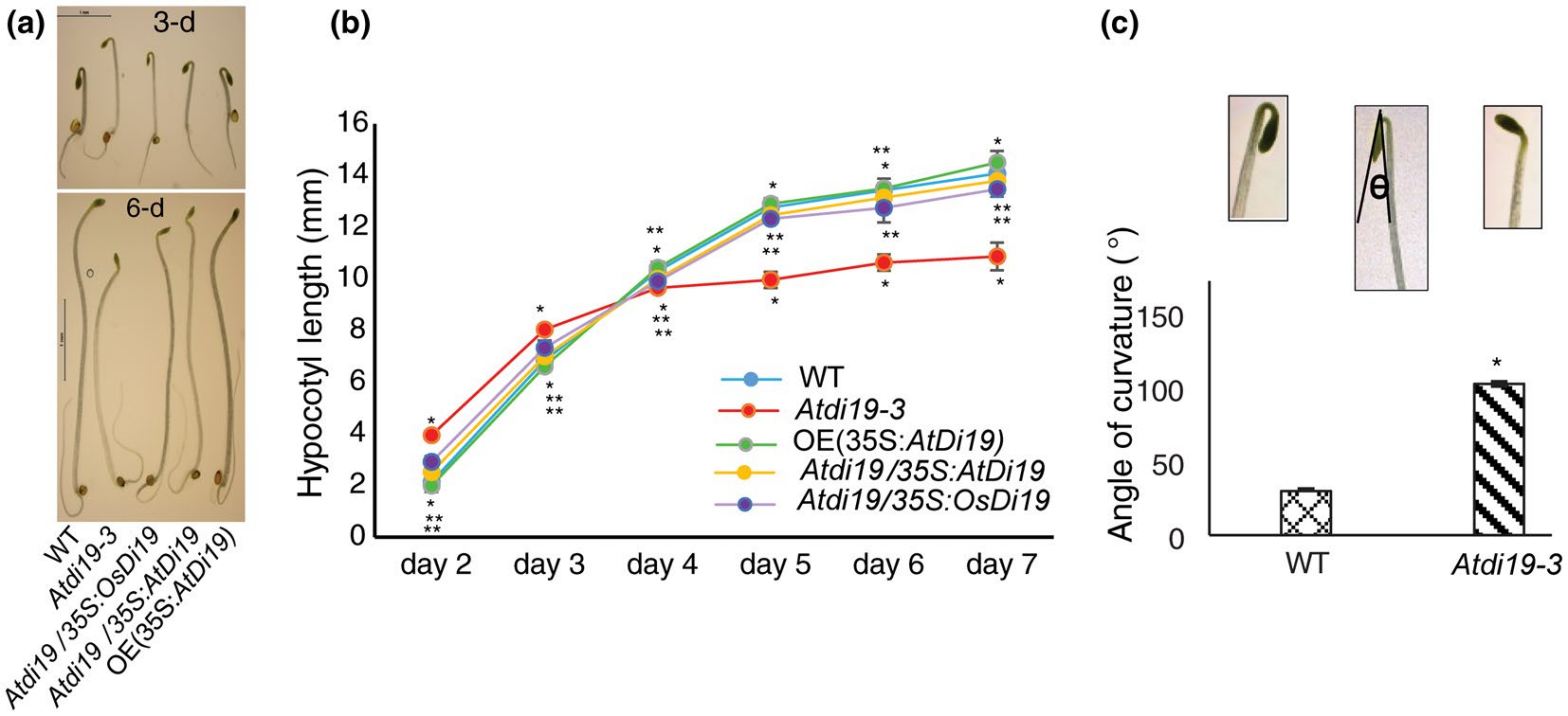
Phenotypic comparison of Arabidopsis *di19* mutant seedlings grown in dark. (a) Phenotype of dark grown seedlings of WT, *Atdi19‐3* mutant, mutant complemented with *OsDi19* and *AtDi19*, and *AtDi19* overexpression line L2. (b) Hypocotyl length of Arabidopsis seedlings, as listed in (a), grown in dark up to 7 days. Mean value ± SE of three independent experiments with 20 seedlings in each set was plotted. (c) Apical hook angle of etiolated seedlings of both the mutant and the wild‐type measured after 60 hr of growth in dark. Mean value ± SE of three independent experiments with 20 seedlings in each set was plotted. One‐way ANOVA was performed followed by Student's *t* test (both at *p* < .05) for estimation of statistical significance. (*wild‐type vs. mutant/overexpressing lines; **mutant versus. complementation lines)

When grown at higher temperature in light, the wild‐type seedlings were more responsive to temperature‐induced hypocotyl elongation in comparison to the *Atdi19‐3* mutant seedlings (Figure 2b); the hypocotyls of the wild‐type seedlings were longer when grown at 28°C instead of 22°C. However, the *Atdi19‐3* mutant seedlings also showed increase in hypocotyl length at 28°C but to a lesser extent than the wild‐type. The 3‐day‐old dark grown seedlings of *Atdi19‐3* mutant were taller than the wild‐type (Figure 3a,b), whereas the hypocotyl of overexpressing lines was somewhat shorter than the wild‐type. Interestingly, when the growth of wild‐type and mutant seedlings was monitored over a period of 7 days in dark, the 3‐day‐old mutant seedlings initially showed elongated hypocotyl as compared to the wild‐type seedlings but eventually the wild‐type seedlings surpassed the growth of *Atdi19‐3* mutant seedlings and developed longer hypocotyls (Figure 3a,b). After 60 hr of germination in dark, it was observed that the apical hook in mutant seedlings is relatively open, whereas the apical hook is still maintained tightly in the wild‐type seedlings (Figure 3c). These observations indicate that *AtDi19‐3* plays an important role in at least some of the auxin‐mediated responses in Arabidopsis.

### 2.3 Higher IAA content in *AtDi19‐3* mutant seedlings

**FIGURE 4:**
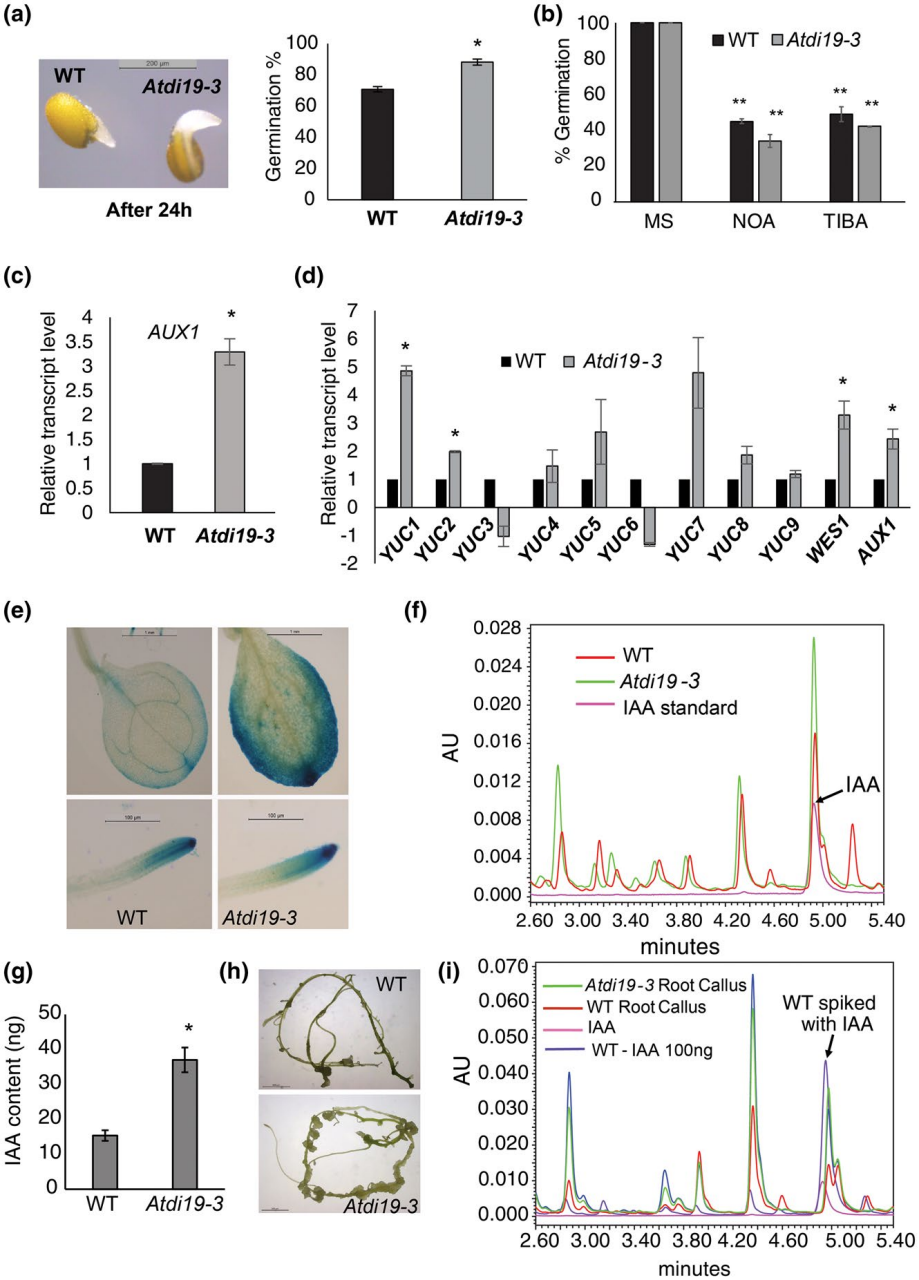
Seed germination assay, differential expression of genes associated with auxin signaling in *Atdi19‐3* mutant and estimation of IAA in wild‐type and *Atdi19‐3* mutant plants. (a) Seed germination in the wild‐type and *Atdi19‐3* mutant on 1/2 MS; (b) Seed germination in the wild‐type and the *Atdi19‐3* mutant in the presence of auxin transport inhibitor NOA (25 µM) and TIBA (25 µM). Germination rate was normalized to that of wild‐type and mutant seeds under untreated conditions. Mean value ± SE of three independent experiments having 30 seeds in each was plotted. One‐way ANOVA was performed followed by Student's *t* test (both at *p* < .05) for estimation of statistical significance (*wild‐type versus. mutant, **untreated vs. treated). (c) *AUX1* transcript levels in dry seeds estimated by RT‐qPCR; (d) Relative transcript levels of *YUCCA* genes in 10‐day‐old light grown seedlings analyzed by RT‐qPCR. Error bars indicates standard error (±SE). One‐way ANOVA showed significant statistical difference; *represents *p* < .05. (e) GUS activity in 10‐day‐old seedlings of *DR5::GUS/Adi19‐3* lines. Note higher expression of DR5::GUS reporter activity in cotyledon and root in seedlings harboring *DR5::GUS* in *Atdi19‐3* mutant background (right panel). Left panel shows GUS induction in *DR5::GUS/*Col‐0 line. (f) Chromatogram of the extract from 2‐week‐old Arabidopsis seedlings as resolved by UPLC (for details, see protocol described in Materials and Methods section). (g) Free IAA content in Arabidopsis seedlings as measured by UPLC. Mean value ± SE of three independent experiments was plotted. One‐way ANOVA was performed followed by Student's *t* test (both at *p < .05) for estimation of statistical significance. (h) Root explants of 2‐week‐old seedlings kept on callus induction medium. (i) Chromatogram showing free IAA content in callusing mutant root explants as measured by UPLC. RT‐qPCR, real time‐quantitative PCR

It has been reported earlier that auxin plays a role in seed germination (Liu et al., 2007, 2013; Wang et al., 2016). In the *Atdi19‐3* mutant, seed germination was also faster as compared to the wild‐type (Figure 4a). The role of auxin in germination of *AtDi19‐3* mutant seeds was further analyzed by imbibing the seeds in the presence of auxin transport inhibitors. There was a decline in the germination rate for both the wild‐type and *AtDi19‐3* mutant seeds but the effect of auxin transport inhibitors, NOA and TIBA, on the suppression of seed germination was significantly greater in the mutant than the wild‐type (Figure 4b). Furthermore, we observed higher transcript levels of gene encoding auxin influx transporter, *AUX1* in *Atdi19‐3* dry seeds (Figure 4c).

To find out whether the auxin‐associated phenotypes displayed by *Atdi19‐3* mutant seedlings are due to any change in auxin levels, we adopted a two‐pronged approach. First, auxin‐responsive reporter *DR5::GUS* was introduced into the *Atdi19‐3* mutant background (see Materials and Methods). Enhanced DR5::GUS activity was observed in the cotyledons and roots of the 10‐day‐old *Atdi19‐3* seedlings in comparison to the wild‐type (Figure 4e; Figure S5a–c). Hence, we quantified IAA in 2‐week‐old light grown Arabidopsis seedlings using UPLC. The UPLC data revealed that the IAA level in *Atdi19‐3* seedlings is more than two‐fold higher than in the wild‐type seedlings (Figure 4f,g).

To examine the sensitivity of explants from *Atdi19‐3* plants toward auxin, wild‐type and mutant seeds were grown in light for 2 weeks, and root explants excised and grown on 1/2 MS medium supplemented with 2,4‐D and kinetin. *Atdi19‐3* mutant root explants formed profuse callus in comparison to the wild‐type root explants (Figure 4h). This result indicated that either the mutant has high auxin content or increased sensitivity to auxin in the root explants. Thus, IAA level in the callusing root tissue was also measured and it was indeed higher in the *Atdi19‐3* callusing root tissue than that in the wild‐type (Figure 4i).

Keeping in view that *Atdi19‐3* mutant seedlings have higher auxin content, the transcript levels of some known auxin responsive genes were quantified by RT‐qPCR. The transcript levels of *AUX*, *WES1*, and some *YUCCA* genes were significantly higher in *Atdi19‐3* mutant seedlings as compared to the wild‐type (Figure 4d), although we did not find any significant change in transcript levels of *PIN2*, *AXR2*, *AXR3*, *ARF7*, and *ARF10* genes (Figure S5e).

### 2.4 Control of LR number by *AtDi19‐3*

**FIGURE 5:**
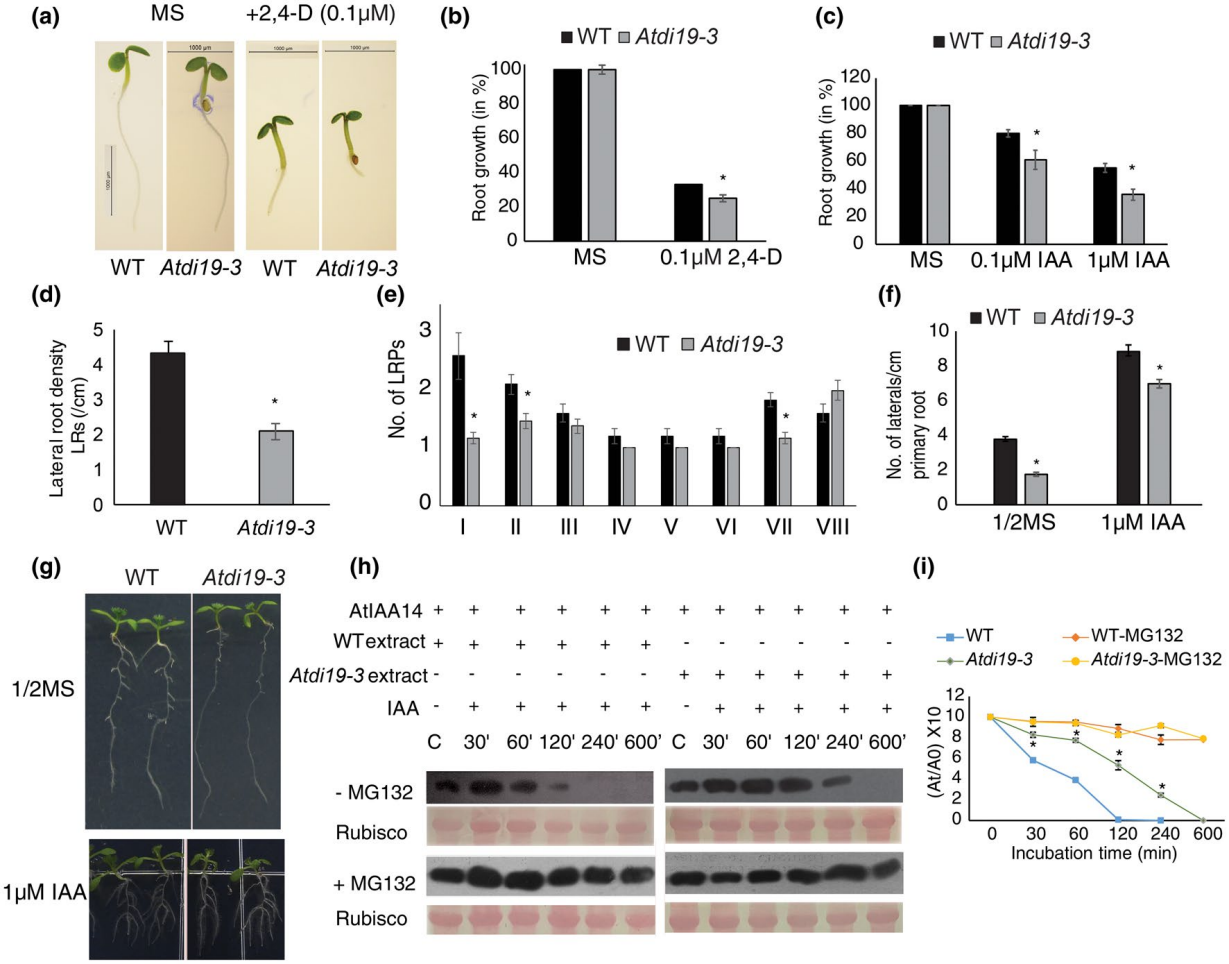
Phenotypic analysis of primary and lateral roots of wild‐type and *Atdi19‐3* mutant plants. (a and b) Root growth of 3‐day‐old seedlings grown on a medium containing 0.1 µM 2,4‐D. Growth was normalized to that of control seedlings grown in the absence of 2,4‐D. Mean value ± SE of three independent experiments having 20 seedlings in each was plotted. (c) Root growth of 5‐day‐old Arabidopsis seedlings transferred to media containing IAA for 3 days. Percentage growth in primary root after IAA treatment was calculated taking the growth of primary root on MS medium as reference. Mean value ± SE of three independent experiments having 20 seedlings in each was plotted. (d and e) Overall lateral root density and staging of lateral root development (from stage I to VIII) in 7‐day‐old *Atdi19‐3* and wild‐type seedlings. Mean value ± SE of three independent experiments having 10 seedlings in each was plotted. Statistical significance (*) tested by Student's *t* test (*p* < .05). (f and g) Lateral root (LR) density in seedlings grown on 1/2 MS media for 2 weeks. Wild‐type and mutant seedlings were grown for 3 days and transferred to 1 µM IAA containing medium for 10 days and the LR density calculated. Mean value ± SE of three independent experiments having 20 seedlings in each was plotted. (h and i) In vitro protein degradation of 6xHis‐AtIAA14 monitored over a period of 600 min using protein extracts from 2‐week‐old seedlings (of both wild‐type and the *Atdi19‐3* mutant) and quantified using imageJ software. The same experiment was carried out in the presence of 50 µM MG132 (protease inhibitor complex) showing that the degradation is indeed 26S proteasome mediated. Mean (±SE) of three independent experiments was plotted. Ponceau stain of the PVDF membrane showing equal loading. One‐way ANOVA was performed followed by Student's *t* test (both at *p* < .05) for estimation of statistical significance (*wild‐type vs. mutant)

The antagonistic effect of auxin on growth of primary root and LRs has been documented earlier (Overvoorde, Fukaki, & Beeckman, 2010; Stepanova et al., 2007; Swarup et al., 2007). In the present study, *Atdi19‐3* mutant seedlings showed increased sensitivity to auxin in primary root growth inhibition assay (Figure 5a–c, Figure S5d). The *Atdi19‐3* mutant seedlings when grown in the presence of 2,4‐D for 3 days showed enhanced inhibition of primary root growth as compared to the wild‐type (Figure 5a,b). Even when grown in 1/2 MS medium for 5 days and then transferred to IAA containing medium, the extent of root growth inhibition by IAA was greater in the *Atdi19‐3* mutant seedlings (Figure 5c; Figure S5d).

When both wild‐type and *Atdi19‐3* seedlings were grown on 1/2 MS medium for 14 days in light, the LR density and number of LR primordia (LRPs) in the mutant seedlings were relatively less (Figure 5d,e). But when the wild‐type and *Atdi19‐3* mutants were subjected to IAA treatment, the number of LRs was found to be higher in both the wild‐type and mutant seedlings (Figure 5f,g), indicating responsiveness of mutant seedlings to exogenous IAA for the emergence of LRs. The LR staging of *Atdi19‐3* showed significant decrease in the number of Stage I and Stage II LRPs compared to wild‐type indicating that the initial stages of LR development are affected significantly in *Atdi19‐3* mutant seedlings (Figure 5d,e; Figure S9). We did not find any significant difference in LR density in the overexpression lines *35S:AtDi19* (L2 and L5) when compared with the wild‐type (Figure S4b).

Our initial study gave an indication of interaction of OsIAA13 with OsDi19‐5 in rice. According to an earlier report on rice gain‐of‐function mutant, *Osiaa13*, the mutant plants displayed auxin‐related phenotype, like reduced LR formation (Kitomi, Inahashi, Takehisa, Sato, & Inukai, 2012). *Osiaa13* has single amino acid mutation in domain II resulting in stabilized OsIAA13 protein, causing reduced LR formation. Phylogenetic analysis has revealed that rice *OsIAA13* and Arabidopsis *AtIAA7*, *AtIAA14*, and *AtIAA16* belong to the same clade (Figure S3). In Arabidopsis, the LR formation is regulated by different Aux/IAA‐ARF modules; one such module represented by SLR/IAA14‐ARF7‐ARF19 has a role in LR initiation and hence LR formation (Fukaki, Tameda, Masuda, & Tasaka, 2002; Goh, Kasahara, Mimura, Kamiya, & Fukaki, 2012; Vanneste et al., 2005). The present study has revealed that AtIAA14 interacts with AtDi19‐3 and *Atdi19‐3* mutants are also compromised in LR density.

According to a study performed on the dynamics of LR development (Guseman et al., 2015), the rate of auxin‐induced IAA14 degradation quantitatively determines the LR development; LR density is directly correlated to the rate at which IAA14 degradation occurs. To assess the turnover rate of AtIAA14 in the *Atdi19‐3* mutant seedlings, we expressed AtIAA14 in *E. coli* BL21 (RIL) cells and purified the protein. The degradation rate of recombinant AtIAA14 was ascertained in the presence of protein extract from 2‐week old wild‐type and *Atdi19‐3* mutant seedlings over a period of 600 min (Figure 5h,i). The rate of AtIAA14 degradation when incubated with protein extract from the mutant seedlings was somewhat slower as compared to the protein extract from the wild‐type seedlings, although it eventually degrades completely when incubated for longer duration. In the above assay, the degradation of AtIAA14 was abolished in the presence of MG132, a proteasome inhibitor (Figure 5h,i), indicating that this degradation is mediated by the 26S proteasome.

**FIGURE 6:**
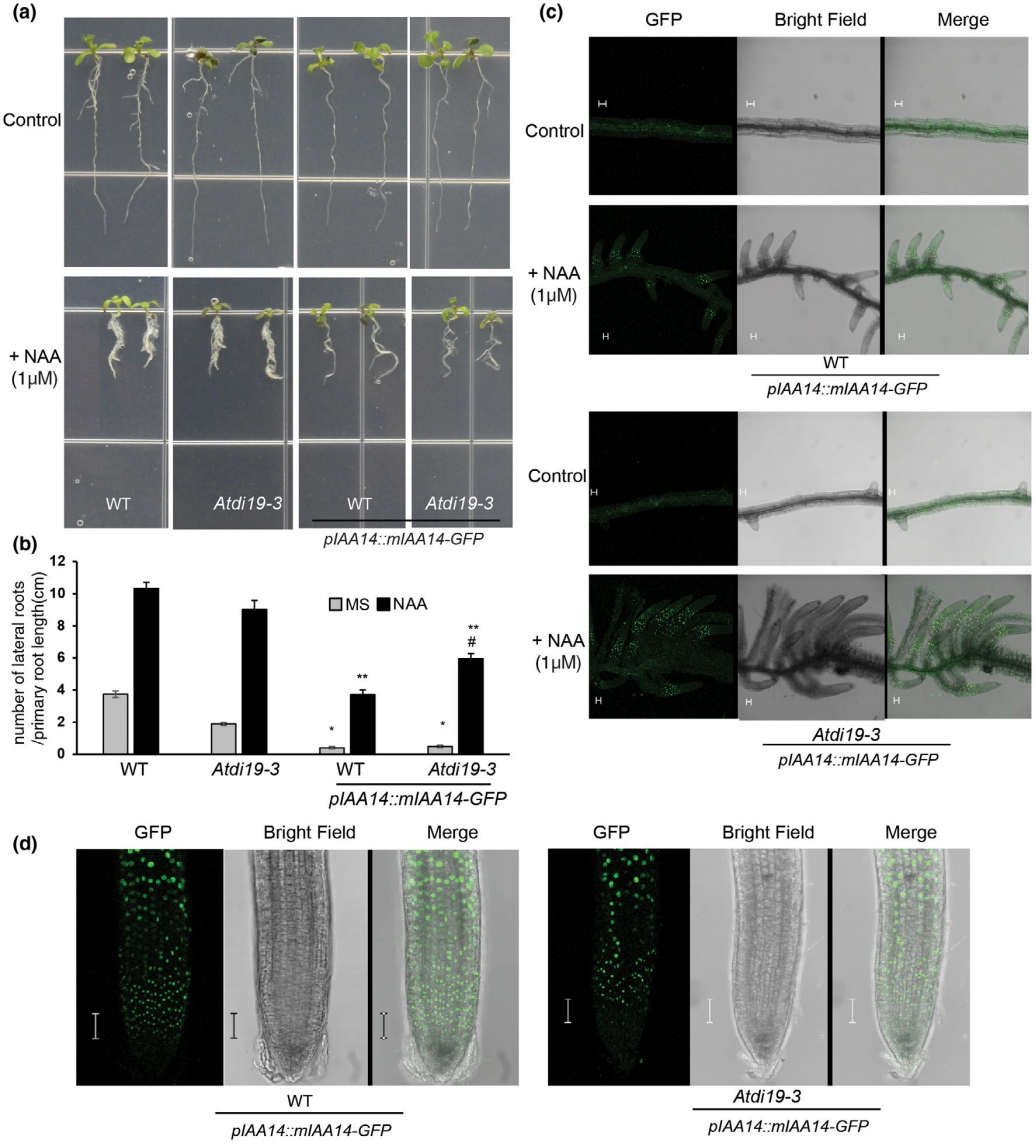
Lateral root formation in *Atdi19‐3* mutant expressing *pIAA14::mIAA14‐GFP*. (a and b) LR density in *Atdi19‐3* mutant and *pIAA14::mIAA14‐GFP* expressing *Atdi19‐3* mutant. Four‐day‐old seedlings were transferred to medium with or without 1µM NAA, and incubated for 72 hr. Mean value ( ± SE) of three independent experiments having 20 seedlings in each was plotted. One‐way ANOVA was performed followed by Student's *t* test (both at *p* < .05) for estimation of statistical significance *represents *p* < .05 (control *di19‐3* vs. control WT or *di19‐3* expressing *mIAA14‐GFP*), **represents *p* < .05 (treated *di19‐3* vs. treated WT or *di19‐3* expressing *mIAA14‐GFP*), ^#^represents *p* < .05 (treated WT with *mIAA14‐GFP* vs. treated *di19‐3* with *mIAA14‐GFP*); (c) Formation of lateral roots in 5‐day‐old wild‐type and *Atdi19‐3* seedlings expressing *pIAA14::mIAA14‐GFP* grown on media with or without NAA; scale‐50 µm. (d) Images of root tip of 5‐day‐old wild‐type and *Atdi19‐3* seedlings expressing *pIAA14::mIAA14‐GFP;* scale‐50 µm. LR, lateral root

To ascertain the behavior of *Atdi19‐3* mutant in the presence of stabilized *mIAA14‐GFP* driven by the *pIAA14* promoter (*pIAA14::mIAA14‐GFP),* we crossed *Atdi19‐3* mutant with Arabidopsis transgenic lines expressing *pIAA14::mIAA14‐GFP* (Figure 6; Figure S6a,b) and raised stable homozygous F3 lines. The LR density in *Atdi19‐3* transgenic lines was further repressed in the presence of stabilized mIAA14‐GFP in comparison to *Atdi19‐3* plants (Figure 6a). When subjected to NAA treatment, the roots of *Atdi19‐3* and *Atdi19‐3* expressing *pIAA14::mIAA14‐GFP* showed LR formation, but *Atdi19‐3* plants showed relatively more LR formation and profuse root hair formation (Figure 6a–c; Figure S6c). The LR density in both wild‐type and *Atdi19‐3* expressing *pIAA14::mIAA14‐GFP* was not significantly different, however, both showed significant difference in LR density in the presence of NAA. The mIAA14‐GFP signal was also detected in the nuclei of both the epidermal and stele cells in roots of both wild‐type and *Atdi19‐3* genetic background (Figure 6d). The GFP signal was less prevalent in cells toward the tip of the root in *Atdi19‐3* seedlings in comparison to the wild‐type, although GFP signal was comparable in the cells of the upper part of the root, away from the root tip (Figure 6d; Figure S6d). Notably, the root tips in *Atdi19‐3* seedlings also showed higher auxin level (Figure 4e). Thus, reduced LR density in the presence of stabilized mIAA14 in *Atdi19‐3* or a delayed degradation of IAA14 in *Atdi19‐3*, both indicate that in all probability, AtDi19‐3 acts as a positive regulator of auxin signaling.

### 2.5 Differential expression of auxin, ethylene, and stress‐associated genes in *Atdi19‐3* and congruous stress response of *slr* mutant

**FIGURE 7:**
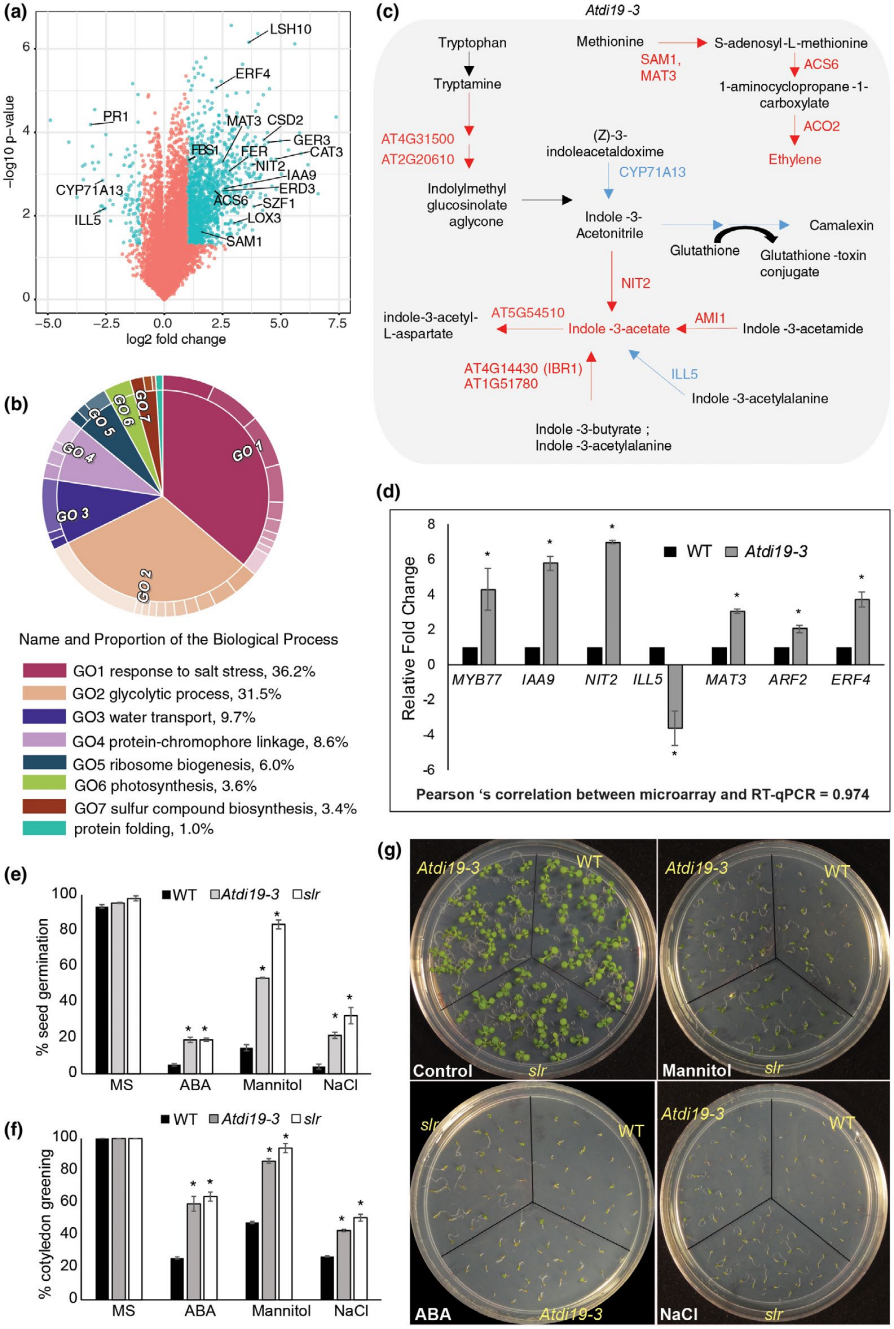
Differential expression of ethylene and stress‐associated genes in *Atdi19‐3* and congruous response of *slr* and *Atdi19‐3* mutant seedlings to abiotic stress. Microarray‐based transcriptome analysis showing differentially expressed genes (fold change >2 or <−2, *p*‐value ≤ .05) in *Atdi19‐3* vs. wild‐type seedlings. (a) Plot showing 721 DEGs in *Atdi19‐3* versus wild‐type (blue dots; Log_2_ fold change >1 or <−1, *p*‐value ≤ .05). Some genes including those identified in pathway analysis have been highlighted. (b) Gene ontology enrichment analysis of differentially expressed genes in microarray experiment; see Figure S11 for detailed analysis. (c) Schematic representation of metabolic pathways enriched among differentially expressed genes in *Atdi19‐3* versus wild‐type. Gene names are colored according to their expression level (red for up and blue for downregulated). (d) Validation by RT‐qPCR for selected differentially expressed genes associated with auxin and ethylene pathways identified through microarray analysis. Error bars indicate standard error (±SE). One‐way ANOVA showed significant statistical difference; *represents *p* < .05. (e) Germination of *Atdi19‐3*, *slr,* and wild‐type seeds were monitored for a period of 7 days on 150 mM NaCl or 300 mM mannitol or 2 µM ABA containing 1/2 MS media. The plot shows seed germination (in %) on day 3 as mean ± SE of three independent experiments with 30 seeds in each. (f and g) Cotyledon assay performed on *Atdi19‐3* and *slr* mutants with respect to wild‐type seedlings. Three‐day‐old seedlings were transferred to 150 mM NaCl or 300 mM mannitol or 2 µM ABA containing 1/2 MS media and were grown for 10 days. (f) Mean values for green cotyledons on 13th day was plotted with error bars ± SE of three independent experiment with 20 seedlings each. (g) Representative picture showing growth of above mentioned seedlings on day 13 under different media compositions. One‐way ANOVA was performed (*p* < .05) followed by Student's *t* test (*p* < .05). *wild‐type versus mutant. DEGs, differentially expressed genes; RT‐qPCR, real time‐quantitative PCR

Transcriptome analysis of 10‐day‐old *Atdi19‐3* mutant and wild‐type seedlings under control conditions using microarray showed that the differentially expressed genes (DEGs) were enriched in KEGG pathways such as proteasome, photosynthesis, glyoxylate metabolism, and superoxide radical degradation (File S2 and File S3). In consonance with an earlier report claiming that Di19 acts as a transcriptional regulator of pathogenesis‐related (PR) genes in Arabidopsis (Liu et al., 2013), we found *PR1* (*PATHOGENESIS‐RELATED GENE 1*) expression was downregulated in *Atdi19‐3* seedlings (Figure 7a). Gene ontology (GO) analysis showed that the DEGs in *Atdi19‐3* were enriched for biological process terms, such as response to salt stress, glycolytic process and water transport, and molecular function terms like mRNA binding, copper ion binding, structural constituent of ribosome and protein binding (Figure 7b, Figure S11). Genes associated with either oxidative stress response (*CSD2*, *CAT3*, *FSD2*), dehydration stress (*ERD3*, *FBS1*, *SZF1*) or auxin signaling (*IAA9* and *IAA8*) were upregulated in *Atdi19‐3* mutant seedlings (Figure 7a). We also observed perturbation in the expression of genes associated with IAA biosynthesis and homeostasis involving pathway components like indole‐3‐acetamide, Indole‐3‐acetonitrile, indole‐3‐acetaldoxime, and indole‐3‐acetylalanine (Figure 7c, File S2 and File S3). Recently, glucosinolate breakdown products have also been implicated in the root development, their levels being regulated by Aux/IAA proteins under drought stress in Arabidopsis (Katz et al., 2015; Salehin et al., 2019). In *Atdi19‐3* mutant seedlings, genes associated with the pathway involving indolylmethyl glucosinolate aglycone biosynthesis from tryptophan were upregulated (Figure 7c). Some of the genes associated with auxin biosynthesis and signaling, such as *NIT2*, *ILL5*, *IAA9*, *MYB77*, *ARF2*, whose expression were found to be altered in *Atdi19‐3* mutant seedlings in the microarray profile, were validated for their transcript levels by RT‐qPCR (Figure 7d). The transcriptome analyses thus provides support to *AtDi19‐3* function in auxin‐mediated responses as well as greater insight into its role under stress as *Atdi19‐3* mutant shows higher drought‐tolerance and reduced ABA sensitivity (Figure S7; present study; Qin et al., 2014).

Lateral root development is a key morphogenic response that links auxin with salt or osmotic stress as LR production under salt stress is reduced in auxin‐related mutants like *axr1*, *axr4*, and *aux1* (see Sharma et al., 2015). Since Di19 protein is known to be associated with stress response and the present study identified its interaction with IAA14, it was prudent to find out how the *slr‐1/iaa14* (*slr*) mutant seedlings respond to abiotic stress conditions. Gain‐of‐function *slr‐1/iaa14* mutant has a stabilized IAA14 protein and completely lacks LRs (Fukaki et al., 2002). The *slr* mutant seedlings showed reduced hypocotyl length, cotyledon size, like *Atdi19‐3* mutants, when compared to wild‐type seedlings, although they lack any LR formation unlike *Atdi19‐3* seedlings (Figure S10). Moreover, *slr* mutant seedlings have small leaves and hypocotyl showed altered cell elongation profile, as reported by Fukaki et al. (2002). The *slr* and *Atdi19‐3* seeds showed comparable germination percentage in media containing ABA, mannitol, and NaCl (Figure 7e). Cotyledon greening assay in *slr* mutant under different stress conditions was also performed and its response was essentially comparable to that of *Atdi19‐3* seedlings (Figure 7f,g). Thus, the data presented above suggest that both AtDi19‐3 and AtIAA14 play a role in abiotic stress response.

### 2.6 Impaired apical hook development in *Atdi19‐3* mutant is indicative of altered hormone response

**FIGURE 8:**
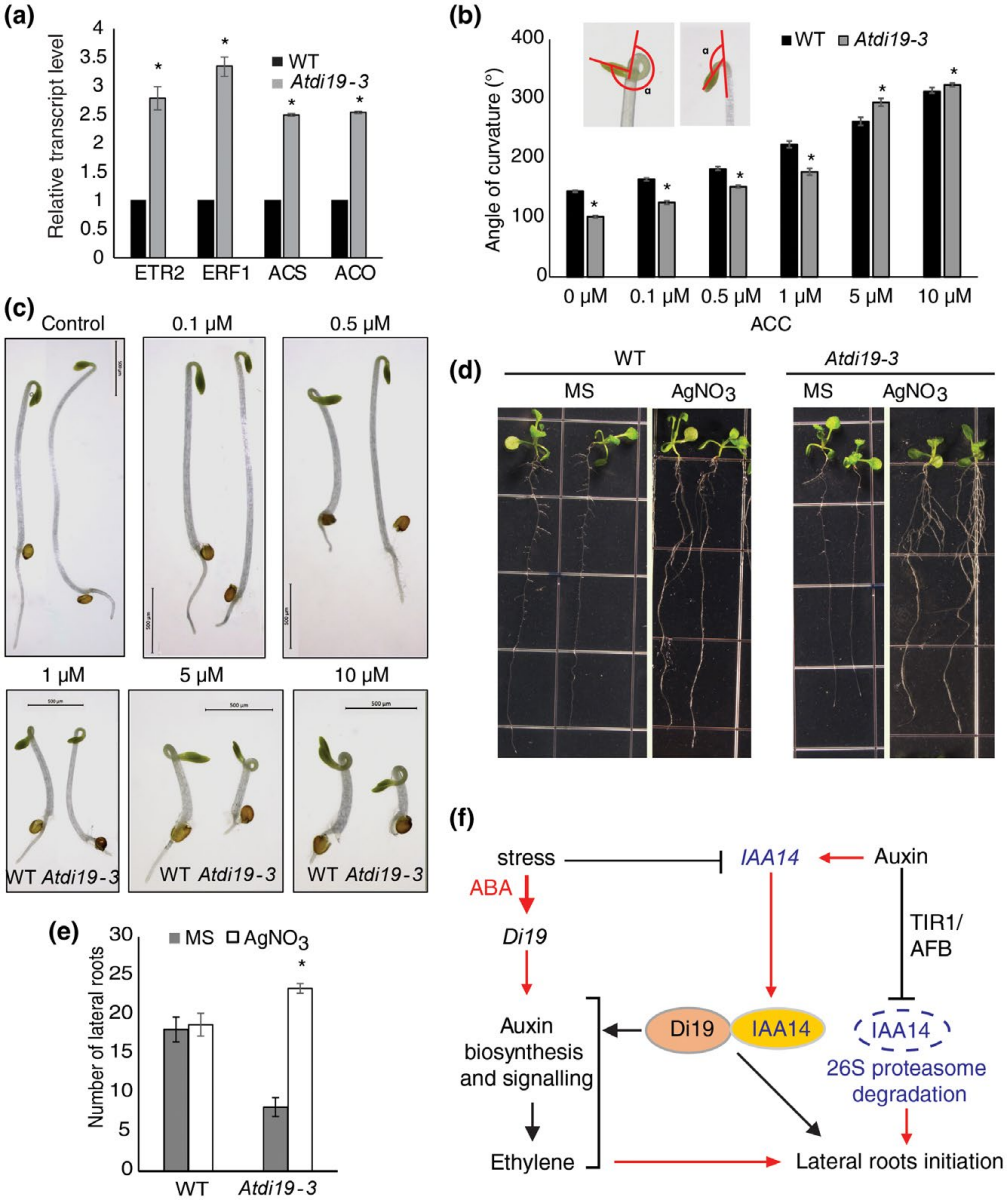
Role of Di19‐3 in ethylene signaling and proposed model for interactive effect of IAA14 and Di19‐3 in auxin signaling. (a) Relative transcript level of ethylene‐associated genes *ETR2*, *ERF1*, *ACS*, and *ACO* in *Atdi19‐3* and wild‐type seedlings. Error bars indicate standard error (±SE). One‐way ANOVA showed statistical significance; *represents *p* < .05. (b and c) Wild‐type and *Atdi19‐3* seedlings grown in the presence of different concentrations of ACC for 3 days in dark. The triple response is distinctly visible in both wild‐type and mutant seedlings; however, the *Atdi19‐3* seedlings are hypersensitive at higher concentrations of ACC. Histogram showing angle of curvature of the apical hook in etiolated seedlings of both the mutant and the wild‐type measured after 3 days. Data represent mean value ± SE of three independent experiments with 20 seedlings in each set. One‐way ANOVA was performed followed by Student's *t* test (both at *p* < .05) for estimation of statistical significance (*wild‐type versus mutant). (d) Seedlings of wild‐type and *Atdi19‐3* mutant grown vertically for 5 days in light and transferred to 10 µM AgNO_3_ containing medium for additional 5 days. (e) Number of LRs measured after 10‐day growth. Data represent Mean ± SE of three independent experiments with 20 seedlings each. One‐way ANOVA was performed followed by Student's *t* test (both at *p* < .05) for estimation of statistical significance; *represents control versus treated. (f) Schematic representation of the hypothesized mechanism. This representation shows the involvement of Di19‐3 in auxin signaling and its role in interaction between auxin and ethylene pathways under drought stress. The expression of *Di19‐3* is induced upon drought stress and ABA. Under drought stress, the expression of *IAA14* is downregulated in Arabidopsis (Shani et al., 2017). Furthermore, IAA14 physically interacts with Di19‐3 and its degradation is delayed in *Atdi19‐3* in the presence of auxin. Physiologically, Di19‐3 may promote stress response such as lateral roots initiation by interacting with IAA14. Several genes whose expression is altered in *Atdi19‐3* seedlings indicate the involvement of Di19‐3 in stress response, auxin, and ethylene biosynthesis as well as signaling pathways (see text for details)

In the transcriptome analysis described above, the expression of genes (*SAM1*, *MAT3*, *ACS6*, and *ACO2*) involved in ethylene biosynthesis from its precursor methionine was upregulated in *Atdi19‐3* mutant (Figure 7a,c). Their transcript levels along with those involved in ethylene signaling response (ETR2, ERF1, and ERF4) were validated by RT‐qPCR wherein the transcript levels of most of these genes were found to be more than two‐fold higher in *Atdi19‐3* mutant as compared to the wild‐type (Figures 7d and 8a).

The interactive effect of ethylene and auxin in manifestation of various developmental processes, like maintaining apical hook and root development in the young dark grown seedlings is well known. These observations led us to assess the effect of ethylene on *Atdi19‐3* mutant seedlings. Ethylene has a major role to play in the maintenance of apical hook in dark. However, 3‐day‐old dark grown seedlings of *Atdi19‐3* displayed not so prominent apical hook. In order to examine the effect of ethylene on apical hook formation, both wild‐type and *Atdi19‐3* mutant seeds were germinated and allowed to grow in the presence of ethylene precursor ACC in dark. Although at lower ACC concentration the response of *Atdi19‐3* was not striking, the *Atdi19‐3* mutant seedlings showed hypersensitivity to ACC at higher ACC concentrations (Figure 8b,c). Normally, the hypocotyls of 3‐day‐old dark grown *Atdi19‐3* mutant seedlings were longer and slender as compared to wild‐type seedlings but in the presence of 5 and 10 µM ACC, the inhibition in hypocotyl growth and apical hook tightening was quite remarkable in the *Atdi19‐3* mutant in comparison to wild‐type seedlings.

The phenotype of ethylene signaling and biosynthesis mutants is well documented (Negi et al., 2008; Stepanova et al., 2007; Swarup et al., 2007; Wang, Li, & Ecker, 2002). We examined the behavior of seedlings in the presence of ethylene antagonist, AgNO_3_ (Negi et al., 2008). In response to AgNO_3,_ the *Atdi19‐3* mutant seedlings showed considerable increase in the number of LRs, whereas wild‐type seedlings showed slight, although statistically insignificant increase in the number of LRs (Figure 8d,e); as stated earlier too, the number of emerged LRs in mutant seedlings was less when grown on basal 1/2 MS medium. Since AgNO_3_ also inhibits aquaporins, increased LR number in *Atdi19‐3* upon aquaporin inhibition can partly explain the resistant behavior of *Atdi19‐3* mutant under water deficit conditions (Figure S7; present study). These observations presented above are indicative of alteration in hormonal interaction that is manifested eventually in the altered phenotypic behavior of the *Atdi19‐3* mutant.

## 3 DISCUSSION

Involvement of Cys2/His2 (C2H2) zinc finger transcription factor family in regulating the plant's response to abiotic and biotic stress has been reported previously (Huang et al., 2009; Kim et al., 2001; Mukhopadhyay, Vij, & Tyagi, 2004; Sakamoto et al., 2004; Xu, Wang, & Chen, 2007). Di19 has atypical ZZ‐type zinc finger motif that has the potential for protein–protein interaction (Kang, Chong, & Ni, 2005) and contains putative nuclear localization signals (NLS). The expression of *Di19* genes is induced upon dehydration and high salinity stress (Milla et al., 2006; Wang et al., 2014). *AtDi19‐1* and *AtDi19‐3* transcript levels increased in Arabidopsis plants subjected to dehydration stress, whereas those of *AtDi19‐2* and *AtDi19‐4* were upregulated by high salinity stress (Liu et al., 2013; Qin et al., 2014). In rice, *OsDi19‐3* and *OsDi19‐4* were induced both by salt stress and dehydration (Wang et al., 2014). In contrast, *AtIAA14* was also found to be downregulated by DREB and AP2 class of TFs under desiccation stress (Shani et al., 2017). In addition, *PR1*, *PR2*, and *PR5* expression is modulated by AtDi19‐1 on dehydration stress (Liu et al., 2013); drought stress induces the expression of At*Di19‐1* that in turn leads to enhanced expression of *PR* genes. In the present study too, *PR1* was downregulated in *Atdi19‐3* mutant (Figure 7a). Moreover, Di19 interacts with Ca^+2^‐dependent protein kinase that is a prerequisite for its trans‐activity (Milla et al., 2006; Rodriguez Milla et al., 2006). Studies have shown that it is the phosphorylated form of Di19 that plays a role in high salinity stress and ABA signaling (Qin et al., 2014, 2016; Wang et al., 2014; Wang, Yu, Xu, Zhu, & Huang, 2016). It has also been reported that *AtDi19‐3* may be involved in drought and salt stress response in ABA‐dependent pathway (Qin et al., 2014). We also observed *Atdi19‐3* mutant to be less sensitive to ABA‐mediated inhibition of seed germination (Figure S7). The young seedlings of *IAA14* mutant (*slr*) also behaved in a manner similar to *Atdi19‐3* seedlings under different abiotic stress conditions (Figure 7e–g). In an earlier study, it has been shown that the overexpression of cotton *GhDi19‐1* and *GhDi19‐2* genes in Arabidopsis confers hyper‐sensitivity to high salinity and ABA (Li et al., 2010). In rice, however, although the overexpression of *OsDi19‐4* conferred drought tolerance to plants but these transgenic plants were hypersensitive to ABA (Wang et al., 2014, 2016).

The Di19 family members are also involved in pathways other than those associated with stress response. For example, the expression of *AtDi19‐7,* also called *HRB1*, is regulated by light (Kang et al., 2005). In the present study, Arabidopsis *Atdi19‐3* mutant seedlings developed short hypocotyls when grown in light. However, when grown in dark, the hypocotyl elongation of *Atdi19‐3* mutant seedlings was more than that of the wild‐type till 3‐day stage of seedling development but the growth was virtually arrested thereafter and that of the wild‐type seedlings was sustained at least for the duration of the experiment. As a result, the 7‐day‐old wild‐type seedlings were much taller than the *Atdi19‐3* mutant seedlings. It has been shown that when IAA is supplied exogenously to the wild‐type seedlings grown in light, hypocotyl growth is reduced. It has been postulated that auxin level in the hypocotyl of wild‐type seedlings is optimal for its elongation and any additional auxin is inhibitory (Collett, Harberd, & Leyser, 2000). Faster seed germination in *Atdi19‐3* could partly explain the faster hypocotyl growth observed initially for 3 days, however, virtual decline in *Atdi19‐3* hypocotyl elongation growth thereafter, as compared to wild‐type, also points toward some developmental changes occurring in the due course. When the growth of wild‐type and *Atdi19‐3* seedlings grown in light was compared, the hypocotyl length of the mutant was found to be shorter. The *Atdi19‐3* mutant plants are less responsive to temperature‐induced changes in hypocotyl elongation growth in comparison to the wild‐type plants. It has been previously reported that hypocotyl elongation at higher temperature is dependent on auxin. The seedlings of mutants of auxin receptor *tir1* are less responsive to temperature‐induced hypocotyl elongation (Gray, Ostin, Sandberg, Romano, & Estelle, 1998). The apical hook in 3‐day‐old etiolated *Atdi19‐3* seedlings was found to be loose as compared to the wild‐type. The hookless phenotype has been observed in auxin over‐accumulating mutants and also in mutants with altered auxin response (Abbas, Alabadí, & Blázquez, 2013). Auxin levels were indeed high in the callusing root explants derived from the *Atdi19‐3*. Moreover, the *DR5::GUS* harboring lines of *Atdi19‐3* seedlings also exhibited more *GUS* activity in the root tip.

In the present study, we also found that Arabidopsis Di19‐3 physically interacts with AtIAA14 and the degradation kinetics of AtIAA14 in *Atdi19‐3* mutant seedling extract is slowed down to some extent (Figure 1; Figure 5h,i). The degradation rate of Aux/IAAs is also influenced by the transcriptional complex between Aux/IAAs and ARFs (Guseman et al., 2015; Korasick et al., 2014). In Arabidopsis, SLR/IAA14‐ARF7‐ARF19 module is one such module that is involved in LR emergence and density (Lavenus et al., 2013; Okushima, Fukaki, Onoda, Theologis, & Tasaka, 2007). Multiple Aux/IAAs are co‐expressed during LR formation. Slow degradation rate of IAA14 may aid the other persistent protein(s) to compete for binding with TIR1‐auxin complex or ARFs (Guseman et al., 2015). Thus, we speculate that binding of IAA14 with Di19‐3 favors the SLR/IAA14‐ARF7‐ARF19 module that contributes to IAA14 degradation and eventually promotes LR formation, although it needs further experimental validation. In the absence of Di19‐3, IAA14 may face competition from other Aux/IAAs that in turn affects its degradation rate. Thus, the phenotype displayed by *Atdi19‐3* mutant seedlings, its response to auxin, and the interaction of Di19‐3 with AtIAA14, indicate that Di19‐3 plays a role in auxin‐mediated plant development.

Although, the root elongation in *Atdi19‐3* mutant seedlings was more strongly inhibited by 2,4‐D and IAA as compared to wild‐type (Figure 5a–c), however, the number of LRs in *Atdi19‐3* mutant seedlings was relatively less under control conditions (on basal medium, without any auxin supplementation). But when subjected to exogenous IAA treatment, LR formation was enhanced in both wild‐type and mutant seedlings (Figure 5d–g). It is worth mentioning here that IAA8, which is involved in LR formation (Arase et al., 2012), was upregulated in the *Atdi19‐3* mutant. Moreover, the root phenotype displayed by the seedlings could also be explained by the combined effect of ethylene and auxin (Figures 5 and 8). The *AUX1* gene expression is altered in the *Atdi19‐3* mutant seedlings (present study) and previous reports also suggest that AUX1 plays a critical role in ethylene regulated root development, thus linking auxin and ethylene signaling pathways for their possible role during root development. Ethylene promotes polar IAA transport through AUX1 resulting in negative effect of ethylene on LR formation (Negi et al., 2008). However, IAA can reverse the negative effect of ethylene on LR formation. Although both ethylene and auxin affect primary root elongation in a similar manner, but auxin and ethylene act antagonistically for LR development (Lewis et al., 2011; Negi et al., 2008). In Arabidopsis, SLR/IAA14‐ARF7‐ARF19 module plays a major role in LR emergence and density (Lavenus et al., 2013; Okushima et al., 2007). The gain‐of‐function mutation in *IAA14* (*slr‐1*) leads to complete loss of LR formation (Fukaki et al., 2002; Ito et al., 2016). In the present study also, we observed that the auxin responsiveness of *Atdi19‐3* transgenics expressing *pIAA14::mIAA14‐GFP* (stabilized mIAA14‐GFP protein) is compromised when compared with *Atdi19‐3* seedlings (Figure 6a–c; Figure S6c); expressing *pIAA14::mIAA14‐GFP* in *Atdi19‐3* mutant background resulted in increased repression of LR formation in comparison to *Atdi19‐3* (Figure 6). The expression of *mIAA14‐GFP* was detected more at the region away from the root tip and not at the root tip in *Atdi19‐3* (Figure 6d; Figure S6d). Although these observations do suggest involvement of Di19‐3 in Aux/IAA‐mediated auxin signaling pathway, whether there are other factors that facilitate this interaction remains to be elucidated.

Difference in auxin level between the wild‐type and *Atdi19‐3* mutant seedlings prompted us to find out which genes are probably responsible for elevated auxin content. Microarray analysis showed that *NIT2* is upregulated in *Atdi19‐3* mutant by almost seven‐fold; it was also validated by RT‐qPCR (Figure 7c,d). Incidentally, NIT2 is involved in IAOX‐dependent auxin biosynthesis pathway, which is *Brassicaceae* species‐specific pathway among plants (Mano & Nemoto, 2012). *YUCCA* gene family encodes flavin monooxygenases that are key enzymes in auxin biosynthesis (Cheng, Dai, & Zhao, 2007; Zhao et al., 2001). The expression of some of the *YUCCA* genes in the *Atdi19‐3* mutant seedlings was indeed upregulated when checked using RT‐qPCR (Figure 4d). In many instances, expression of *YUCCA* genes has also been associated with drought tolerance in Arabidopsis (see Sharma et al., 2015). Among those responsible for auxin transport, as mentioned before, we found only *AUX1* coding for an auxin influx transporter to be upregulated by two‐ to three‐fold in *Atdi19‐3* mutant seeds as well as seedlings (Figure 4c,d). Recently, Wang et al. (2016) have shown that transgenic plants overexpressing *AUX1* have an enhanced rate of germination. However, this influence of auxin on seed germination is dose dependent. It may positively or negatively affect the rate of seed germination (Liu et al., 2007, 2013; Wang et al., 2016). Even low level of IAA has been reported to facilitate germination (He et al., 2012). *Atdi19‐3* mutant seeds showed faster germination as compared to wild‐type seeds (Figure 4a), which was significantly reduced in the presence of auxin transport inhibitors, TIBA and 1‐NOA (Figure 4b), substantiating the role of auxin in germination of *Atdi19‐3* seeds.

It has also been documented previously that auxin promotes ACC synthase activity, thus stimulating ethylene production (Hansen & Grossmann, 2000). In *Atdi19‐3*, among other genes for ethylene biosynthesis and signaling response, genes coding for ethylene biosynthetic enzymes ACC synthase and ACC oxidase were upregulated (Figures 7 and 8). The mutant showed sensitivity to ethylene in triple response assay and AgNO_3_, an ethylene inhibitor, caused profuse LR formation in the mutant seedlings (Figure 8d,e). These findings thus support the view that high IAA content influences the ethylene biosynthesis pathway and vice‐versa. Thus, Di19‐3 can be postulated as an important component in balancing the two pathways.

The present study indicates that drought stress‐induced Di19‐3 influences auxin as well as ethylene response pathways in Arabidopsis (Figure 8f). Di19‐3 promotes stress‐related responses, such as production of LRs, by interacting with IAA14, and possibly aids in further processing of auxin response signal through destabilizing the repressor proteins. Expressing stabilized mIAA14 in *Atdi19‐3* mutant background resulted in further reduction of LR formation in comparison to *Atdi19‐3* mutant which also corroborates that IAA14 and Di19‐3 most likely work in the same pathway. Since ethylene also inhibits the number of LRs and promotes seed germination, these phenotypes in *Atdi19‐3* mutant can also be partly attributed to the effect of ethylene. Although we did not focus on ethylene, the overlap between ethylene and auxin signaling is well known. Physiologically, Di19‐3 appears to regulate ethylene levels that are usually induced under stress and repression of the same allows auxin action to be de‐repressed as indicated by promotion of LRs by ethylene antagonist. More work of course needs to be done to decipher the molecular link between Di19‐3, auxin, and ethylene. However, from these observations it can be inferred that interaction of Drought‐induced 19 (Di19‐3) with IAA14 is important for auxin signaling leading to promotion of LRs and maintaining a balance in auxin level in the cellular milieu, which is necessary for plant growth and more so under stress conditions.

## 4 MATERIAL AND METHODS

### 4.1 Plant material, growth conditions, and genetic transformation

The T‐DNA insertion mutant of *At03g05700* (SALK_072390) was obtained from ABRC. Plants were grown in 1/2 MS medium with 1% (w/v) sucrose and 0.8% (w/v) agar in white light (50–60 µmol/m^2^ s^‐1^) under 16 hr light and 8 hr dark cycle, at 22 ± 1°C, after 72 hr of stratification at 4°C. For raising overexpression lines and complementation lines, *AtDi19‐3* and *OsDi19‐5* were cloned in *PMDC32* vector and transformed in wild‐type Col‐0 and *Atdi19‐3* mutant, respectively, using floral dip (Burman, Bhatnagar, & Khurana, 2018). *DR5::GUS/*Col‐0 lines were crossed with *Atdi19‐3* plants. F2 lines were screened for mutant phenotype and then analyzed for *GUS* induction using 5‐bromo‐4‐chloro‐3‐indolyl‐β‐D‐glucuronic acid (Sigma Aldrich) as substrate (Jefferson, 1987). F3 seeds were used for all physiological and molecular analyses. To introduce *pIAA14::mIAA14‐GFP in Atdi19‐3* mutant background, we crossed *Atdi19‐3* mutant with *pIAA14::mIAA14‐GFP* lines. F2 lines were screened for mutant phenotype showing GFP expression and T‐DNA insertion (Figure S6). For ACC treatment, seeds were allowed to grow in different concentrations of ACC (Sigma) for 3 days in dark. All the photographs were taken using a stereo microscope (Leica DFC295) and measurements were done using ImageJ software.

For root inhibition assay, seeds were allowed to germinate in 1/2 MS medium, supplemented with 0.1 µM 2,4‐D (Sigma) for 3 days in light and the primary root length was measured. In another set, 5‐day‐old light grown seedlings were transferred to specified concentrations of IAA containing medium and grown vertically for 3 days. For LRs, 3‐day‐old light grown seedlings were transferred to 1 µM IAA/NAA containing medium and grown for 10 days; following this, number of LRs was counted. For germination assay, seeds were plated on filter paper (spread in 90 mm plates) pre‐soaked in water, TIBA (Sigma) or NOA (Sigma) solution, stratified at 4°C for 72 hr, and then transferred to a growth room maintained at 22 ± 1°C before scoring for germination after defined duration. Radicle emergence was considered as the sign for seed germination.

Cotyledon greening assay was performed where 3‐day‐old wild‐type, *Atdi19‐3* and *slr* seedlings were transferred to 150 mM NaCl, 300 mM mannitol, and 2 µM ABA (Qin et al., 2014) and grown for 10 days. Germination percentage was also assessed in the presence of 150mM NaCl, 300mM mannitol, and 2 µM ABA over a period of 7 days.

### 4.2 Yeast Two‐Hybrid assay

For cDNA library screening, RNA was isolated from 3‐day‐old dark grown coleoptile tissue and cDNA library was made in pGADT7‐rec and transformed in Y187 strain following manufacturer's protocol (Clontech). OsIAA13 was cloned in pGBKT7 and checked for autoactivation (Figure S1a,b). The cells with bait plasmid were allowed to mate with the cDNA library transformed cells and plated on *SD*‐AHLW (+X‐ɑ‐Gal) plates for screening, following manufacturer's protocol (Matchmaker™ Gold yeast two‐hybrid System user manual, Clontech).

*AtDi19‐3* and *OsDi19‐5* clones were subcloned in *pGADT7* (Clontech), whereas *AtIAA14*, *AtIAA16*, and *OsIAA13* were cloned in *pGBKT* (Clontech). Individual constructs were checked for autoactivation prior to performing Y2H experiments (Figure S1a,b). The plasmids were co‐transformed in AH109 cells and selected on *SD*/‐LW media. Interaction between pAD‐GAL‐Di19 and pBD‐GAL‐IAA was analyzed by the expression of *HIS3* reporter gene and *MEL1* reporter gene.

### 4.3 Callus induction

Root explants were excised from 2‐week‐old light grown seedlings and kept in callus induction medium containing 0.25 µM 2,4‐D (Sigma) and 2 µM kinetin (Sigma) for one week (Laxmi, Paul, Raychaudhuri, Peters, & Khurana, 2006) under a daily cycle of 16 hr light and 8 hr dark, in a culture room maintained at 22 ± 1°C. Observations for callus induction were made periodically.

### 4.4 RNA isolation, RT‐qPCR, and microarray analysis

Total RNA was isolated from 10‐day‐old light grown seedlings using Trizol as described earlier (Jain, Nijhawan, Tyagi, & Khurana, 2006). Total RNA from dry seeds was isolated following protocol by Meng and Feldman (2010). One microgram of total RNA was used for cDNA synthesis using ABI High capacity cDNA synthesis kit following manufacturer's protocol. RT‐qPCR was performed using LightCycler^®^ 480 SYBR Green I Master (Roche) in the LightCycler^©^480II Real Time Machine (Roche). Three different control primer sets (*UBQ*, *ACT2*, *UBC*) were taken for relative expression analyses. The experiment was repeated three times taking three technical replicate each time. Mean and standard error were calculated from three different experiments. All the primers used for RT‐qPCR are listed in Table S2. The 10‐day‐old light grown seedlings were processed for microarray analysis. Microarray analysis was performed following Borah et al. (2017) and Sharma, Jain, and Khurana (2019).

### 4.5 Quantification of free IAA

One gram tissue, each of 10‐day‐old light grown wild‐type Col‐0 and *Atdi19‐3* mutant seedlings, was homogenized and IAA was extracted in the solvent containing methanol: isopropanol: glacial acetic acid (20:79:1) overnight at 4°C in dark (Müller & Munné‐Bosch, 2011). All the components were of HPLC grade. Extract was passed through C‐18 Sep‐Pak Cartridge (Waters). Eluates were evaporated at 4°C and reconstituted in 50 µl ammonium formate. UPLC was performed using Waters Acquity UPLC, with the following parameters: Solvent A: 5 mM ammonium formate, pH 4.0, Solvent B: Methanol, Column: Waters BEH C18 2.1 × 50 mm, particle size 1.7 µm, Column Temperature 40°C, Detector: PDA Detector at 260 nm; total run for 12 min. Sample chromatogram was compared with standard IAA (Sigma).

### 4.6 In vitro pull‐down assay and protein degradation assay

Recombinant 6‐X His tagged proteins were expressed in *E. coli* (BL21‐RIL) cells by cloning full length coding sequence of *OsDi19‐5* and *AtIAA14* in pET28a(+) vector (Novagen). Similarly, GST‐tagged recombinant proteins were obtained by cloning the CDS of *OsIAA13* and *AtDi19‐3* in pGEX4T1 vector (Amersham Biosciences) followed by induction in *E. coli* (BL21‐RIL) cells. In vitro pull‐down assay was performed following Kepinski (2009). Bacterial lysate expressing GST, GST‐OsIAA13, and GST‐AtDi19‐3 fusion proteins were immobilized onto GSH‐Sepharose (GE Healthcare) by gently mixing at 4°C for 30 min. Following sedimentation, column was washed thrice in 10 bed volumes of ice cold PBS containing 0.5% Triton X‐100, 1 µM PMSF, and 10 µM DTT (Kepinski, 2009). Bacterial lysate of OsDi19‐5 and AtIAA14 were pre‐cleared by incubation with Sepharose‐4B beads (GE Healthcare). About 4 µg of pre‐cleared protein extracts of 6‐X‐His tagged OsDi19‐5 and AtIAA14 were incubated with the fusion protein immobilized Sepharose 4B beads, respectively, for 1h at 4°C in EB (150 mM NaCl, 100 mM Tris–Cl‐pH 7.5, 0.5% NP40, protease inhibitor, MG132) in the presence of 5 µM IAA and 50 µM MG132. Following this, the immobilized beads were washed thrice with EB. Proteins were eluted from the beads by adding 1X loading buffer for SDS‐PAGE. Proteins were transferred to PVDF membrane (GE Healthcare) and probed with anti‐His Ab (Sigma) and developed with Chemiluminiscence kit (MERCK) following manufacturer's protocol.

In vitro protein degradation assay was carried out following Thakur et al. (2005). Full length sequence of *AtIAA14* was cloned in pET28a (+) vector (Novagen) and expressed in *E. coli* BL21 (RIL) cells and then purified using Ni‐NTA agarose beads following manufacturer's protocol (Qiagen). For in vitro protein degradation assay, plant proteins were extracted from 2‐week‐old light grown seedlings in extraction buffer (200 mM Tris–Cl pH 8.0, 100 mM NaCl, 400 mM sucrose, 10 mM Na_2_EDTA, 14 mM β‐mercaptoethanol, 1 mM phenylmethylsulfonylfluoride, 1% plant protease inhibitor cocktail, 0.05% Tween‐20, and 5% glycerol) followed by incubation on ice for 20 min; debris was then pelleted down. About 40 µg plant protein extract was incubated with 7.5 µg of purified recombinant AtIAA14 in the presence of 5 µM IAA and 50 µM MG132 separately for specified duration. Samples were resolved on SDS‐PAGE and immunoblotting carried out as mentioned previously.

### 4.7 BiFC assay

*OsDi19‐5*, *OsIAA13*, *AtDi19‐3*, and *AtIAA14* were amplified using Phusion high fidelity Taq polymerase (NEB Inc.) and cloned in pENTR/D‐TOPO vector (Invitrogen Inc.) as per the manufacturer's protocol. *OsIAA13* and *AtIAA14* were then transferred to pSITE‐cEYFPC1 and *OsDi19‐5* and *AtDi19‐3* into pSITE‐nEYFPC1 BiFC Gateway vectors (Chakrabarty et al., 2007) using Gateway LR clonase enzyme mix. Thereafter, for particle bombardment, 3–5 µg DNA was used to coat 0.5 mg of gold particle and onion epidermal cells were bombarded with the constructs following the protocol as described earlier (Burman et al., 2018). The onion peels were viewed for YFP expression under confocal microscope (Leica TCS SP5).

### 4.8 Tissue staining

Hypocotyl of 7‐day‐old seedlings were excised and kept in tissue clearing solution (70% lactic acid) for 3–4 days at 50°C with regular changes (Herr, 1993). The tissue was then stained with 0.01% toluidine blue and viewed under fluorescence microscope (Leica DFC450C).

### 4.9 Accession numbers

OsDi19‐5: Os01g73960; OsIAA13; Os03g53150; AtDi19‐3; At3g05700; AtIAA14: At4g14550. The normalized microarray data were submitted to NCBI GEO (GSE104975).

## CONFLICT OF INTEREST

The authors declare no conflict of interest.

## AUTHOR CONTRIBUTIONS

SMM designed and contributed to most of the experiments. SMM, ES, and JPK raised the *Atdi19‐3* mutant lines in *pIAA14::mIAA14‐GFP* background. SMM and ES performed the stress assays. SMM and BS did onion peel bombardment experiments and UPLC analysis. ES and BS did the microarray analysis and ES helped in designing RT‐qPCR experiments. SMM and ES drafted the manuscript. JPK supervised the research work, gave inputs in designing the experiments and finalized the manuscript. All authors read and approved the contents of the manuscript.

## Supporting information

Table S1‐S2‐Fig S1‐S12

File S2

File S3
