# Supplementary material for: Drought‐induced protein (Di19‐3) plays a role in auxin signaling by interacting with IAA14 in Arabidopsis
Source: Plant Direct. 2020 Jun 21;4(6):e00234. doi: 10.1002/pld3.234 (PMC7306619; doi:10.1002/pld3.234)
Supplement: Supplementary file 1 — Table S1‐S2‐Fig S1‐S12 [file PLD3-4-e00234-s001.pdf]

Supplementary
Tables and Figures

	OsDi19-5	OsDi19-3	OsDi19-4	OsDi19-6	OsDi19-2	OsDi19-1	OsDi19-7	AtDi19-7	AtDi19-4	AtDi19-3	AtDi19-6	AtDi19-1	AtDi19-5	AtDi19-2
OsDi19-5		22.7	22.2	49.3	26.7	27	27.4	24.4	24.3	29.2	28	26.9	22.5	22.6
OsDi19-3	42.7		41.6	25.3	35	68.4	36.4	31.1	34	34.3	34.7	29.4	27.2	25
OsDi19-4	42	64.6		26.1	31	47	31.5	30.8	34.7	33.2	29	30.4	28.2	25.4
OsDi19-6	65.9	43.5	44.5		26.4	27.9	26.5	24.7	24.6	27.3	28.3	29.3	26.3	22.2
OsDi19-2	45.9	53.3	57.1	45.9		34.8	38.3	41.9	42.6	41.7	38.5	37.4	28.6	29.4
OsDi19-1	46.5	78	71	46	58.4		34.2	34	34.9	40	37.9	33.6	27.4	26.3
OsDi19-7	44.5	49.2	47.3	47.2	59.7	53.5		37	38.7	36.1	35.8	36.9	30.8	26.6
AtDi19-7	45.5	51.2	51.4	44.5	62.7	58.8	55.5		74.7	46.9	46.7	38.7	35.8	37.4
AtDi19-4	45.5	54.9	58.4	44.6	65.2	62.8	58	82.6		47.9	46.8	42	36.1	39.9
AtDi19-3	48.9	54.1	56.7	50.2	66.5	65	56.5	69.1	72.3		74.9	42.4	34.5	30
AtDi19-6	46.8	54.5	54.7	47.3	67.8	61.9	56.8	68	71.4	87.4		42.3	34.9	31.5
AtDi19-1	50	49.6	51	50	57.5	54	54.6	61.1	64.7	67.7	64		32.1	33.9
AtDi19-5	45.3	48.4	48.6	43.5	51.1	53.5	50.9	59.3	59.8	57.4	57.7	50.9		56
AtDi19-2	44.8	44.7	46.1	43	52.8	51.3	45.2	57.9	57.1	51.6	52.7	50.2	71	

Supplementary Table S1. Percentage identity and similarity of Di19 proteins among Osdi19 homologues in *Arabidopsis*. The identity and similarity was analysed using MaTGAT v2.0 software. BLOSUM50 matrix was used with default values.

Primer	Sequence
72390-LP	TCATATAACCCTTCAGCACGC
72390-RP	TGATTAGTTTGGGGATACCCC
ATDi19-3-RT_F	GCTGCTGACCCATTGTTATCG
ATDi19-3-RT_R	GCATGACTCCGTGGTGAAGA
AtIAA14-F	ATGAACCTTAAGGAGACGG
AtIAA14-R	TGATCTGTTCTTGAACTTCTCCATTG
AtDi19-3-F	GAATTCATGGATTCCGATTCATGGAGTG
AtDi19-3-R	CTCGAGTAAGCTGTCATCAAGAATCGTAG
AUX1-rtF	TTCAGCTGCGCATCTAACCAA
AUX1-rtR	TCTGTATTCGACGTAGAGAACAG
AtIAA14-RTF	TCCCCGGAGGCACTGAA
AtIAA14-RTR	AAGCCTCTCTTGTTCCCAACAC
ETR2-rtF	TTCGAACCGGGCAGTTACAC
ETR2-rtR	AATGGCGGTAAGGCAATCG
ERF1-rtF	TCCTCGGCGATTCTCAATTTT
ERF1-rtR	CAACCGGAGAACAACCATCCT
ACS-rtF	TTAGCTAATCCCGGCGATGG
ACS-rtR	ACAAGATTCACTCCGGTTCTCCA
ACO-rtF	TCTACGTTCGTCACCTCCCTCA
ACO-rtR	CTCTTACCAAAGTCTTTCATGGCC
UBQF	GGTGCTAAGAAGAGGAAGAAT
UBQR	CTCCTTCTTTCGGTAAACGT
Actin2-Fp	GCCATCCAAGCTGTTCTCTC
Actin2-Rp	GCTCGTAGTCAACAGCAACAA
yuc3-rtf	ATTGTGGTGGCAACGGGAGA
yuc3-rtr	CCTTGGTACCTTCCACCGGAT
yuc4-rtf	TGGACCGGTTGTTCACACCA
yuc4-rtr	TCCAAGCTGACCTCCATACCG
yuc5-rtf	ACGCGTGGAAAGGGAAATCG
yuc5-rtr	CGGTGACCCACTCGTGTTCT
yuc6-rtf	GGTTGAGTCGGCTGCGTTTG
yuc6-rtr	TCCGTCGTGCCTTCTTCTCC
yuc7-rtf	GTGGCGGGTCCAGACTGTTT
yuc7-rtr	CCTCAAACTCCGGCACCACT
yuc8-rtf	TGCGGTTGGGTTTACGAGGA
yuc8-rtr	CCTTGAGCGTTTCGTGGGTT
yuc9-rtf	AGCGTTGAGTCTGCTCGGTT
yuc9-rtr	GGACAACACGTTCGGCGTTT
wes1-rtf	GTTCCCTGATCACCGATCCTTCA
wes1-rtr	TCCCTTGCCAGGACGACTTC
yuc1-rtr	AGCTCCGACATCGAGAACCG
yuc2-rtf	ATCCACGAGCCTGCTCAAGT
yuc2-rtr	TGCGTTCAAGAGGGCCAAGT
UBC-rtf	TCAAATGGACCGCTCTTATC
UBC-rtr	CACAGACTGAAGCGTCCAAG
sGFP_F	AAGGGCGAGGAGCTGTTCACC
sGFP_R	TTGTACAGCTCGTCCATGCCGTGA
AtIAA16-F	GCTAGCATGATTAATTTTGAGGCCACGG
AtIAA16-R	AAGCTTACTTCTGTTCTTGCACTTTTCTAATG

MAT3_rtf	TTCAGGCTTCTTGGTCAAAT
MAT3_rtr	CTCTGCTGATGTTGGTCTTG
NIT2_rtf	TATTGTTTCCCAAGGTGGAAGTG
NIT2_rtr	ACATCACCAAGATCAAGATCAGC
IAA9_rtf	AAGACTATGTGCTCACTTATGAGG
IAA9_rtr	ACATCAATAAACATCTCCCACGG
ARF2_rtf	ATGTAGATTTAAAGGCAGAGGCAG
ARF2_rtr	CATTCTCGTCTTGATTAGCCTCAG
ILL5_rtf	GTTATTGGATATGTTGGAACTGGC
ILL5_rtr	CCATTCCACCATTTCCTGAATAGG
ERF4_rtf	GACTGTGCTTCTCCTCCGAC
ERF4_rtr	CGACCTACGTTACCGATCCC
MYB77_rtf	ACCTACTTCGTTGAGTTTGTCAC
MYB77_rtr	CTTCATCTGACTCTCAAATCTCGG

Supplementary Table S2: List of primers used for mutant genotyping, plasmid construction and RT-qPCR.

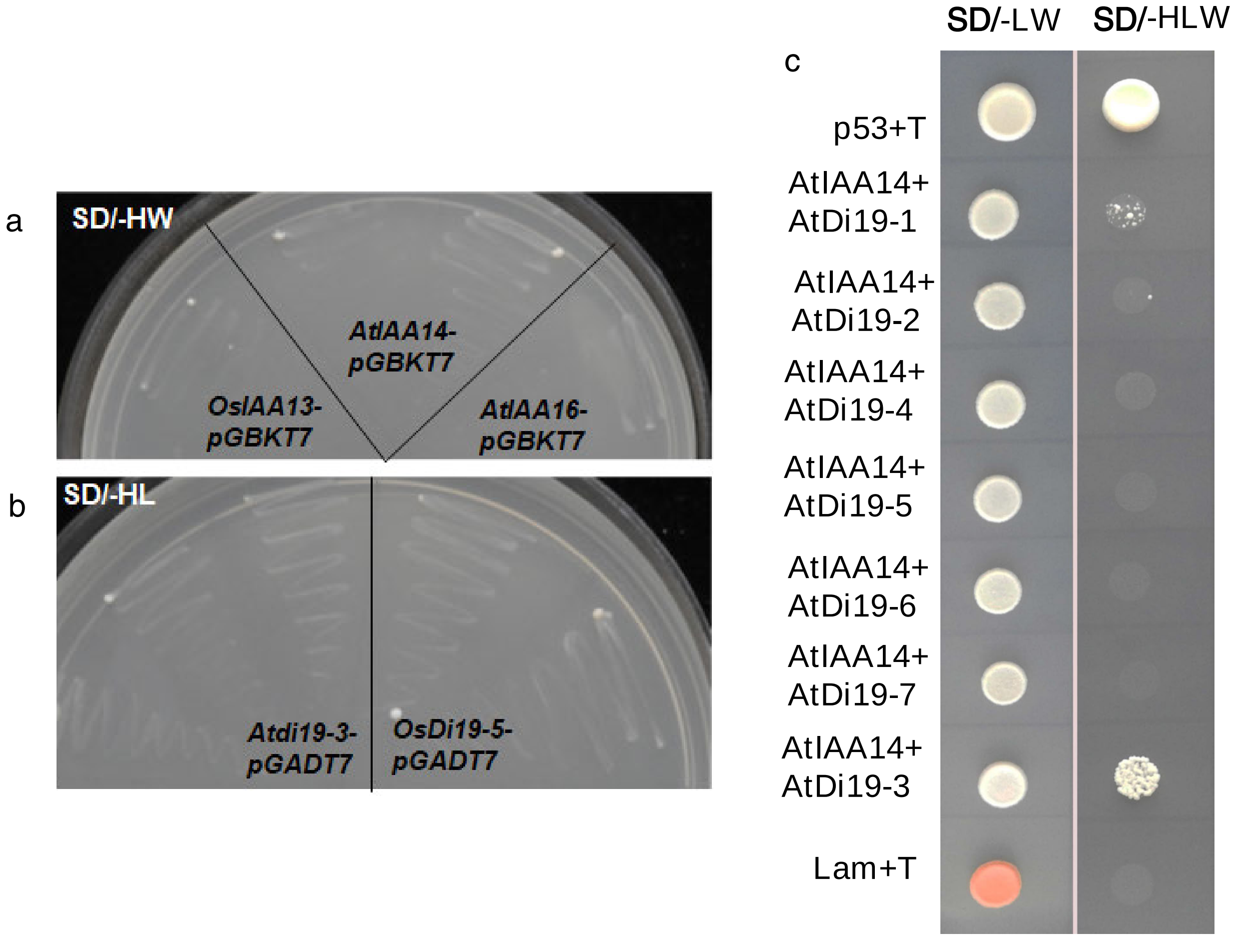

Supplementary Fig S1. (a,b) Autoactivation screening of individual constructs of AtDi19-3-pGADT7, OsDi19-5-pGADT7 in SD/-HL medium and AtIAA14-pGBKT7, AtIAA16-pGBKT7, OsIAA13-pGBKT7 in SD/-HW medium. **(c)**Yeast Two-Hybrid experiment showing interaction among AtDi19 family members and AtIAA14 from *Arabidopsis.*

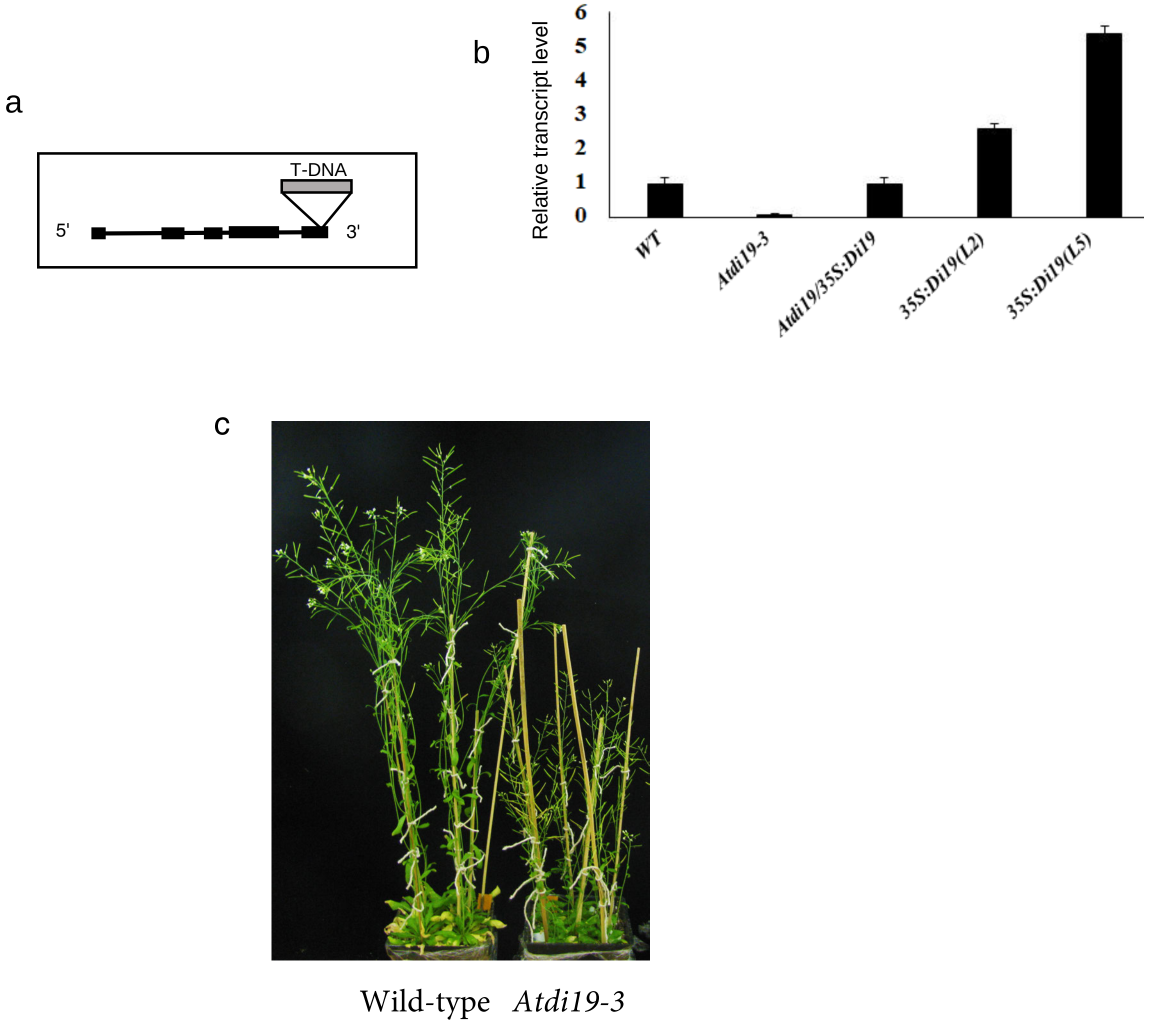

Supplementary Fig S2. Mapping of T-DNA insertion in SALK_072390. **(a)** Schematic representation of T-DNA was verified by running PCR from the genomic DNA of the homozygous line with LBb1.3 and RP and sequencing the PCR product. **(b)** Plot shows relative transcript level values $\pm$ SE for *AtDi19-3*. RNA was extracted from 7-day-old light grown seedlings. Expression of *AtDi19-3* was checked using RT-qPCR. The experiment was done three times with three technical replicates in each. (**c**) *Arabidopsis* plants of wild-type Col-0 and *Atdi19-3* mutant after 1 month under normal conditions(16h/8h light, 22 ºC).

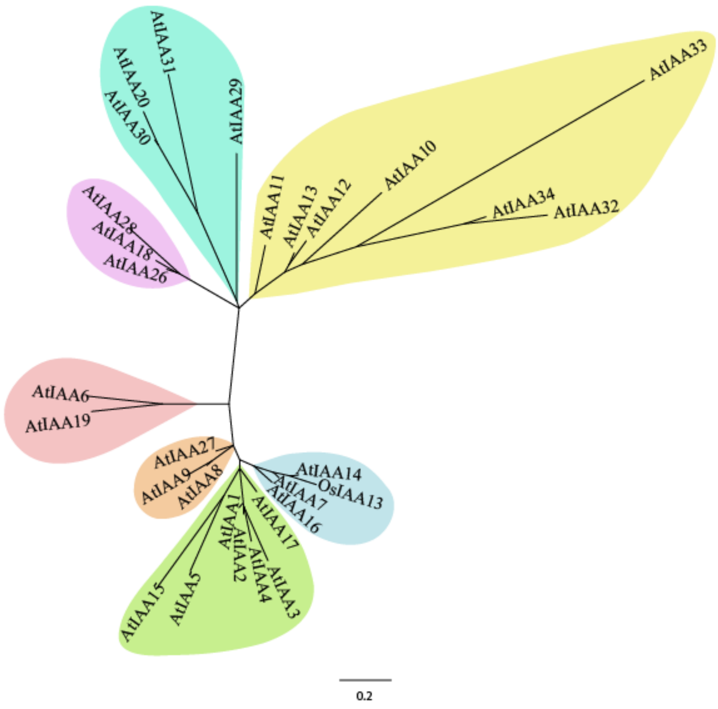

Supplementary Fig S3. Phylogenetic analysis of OsIAA13 in respect to all the AtIAAs. The tree predicts that *AtIAA16*, *AtIAA7 & AtIAA14* are grouped together in one clade.

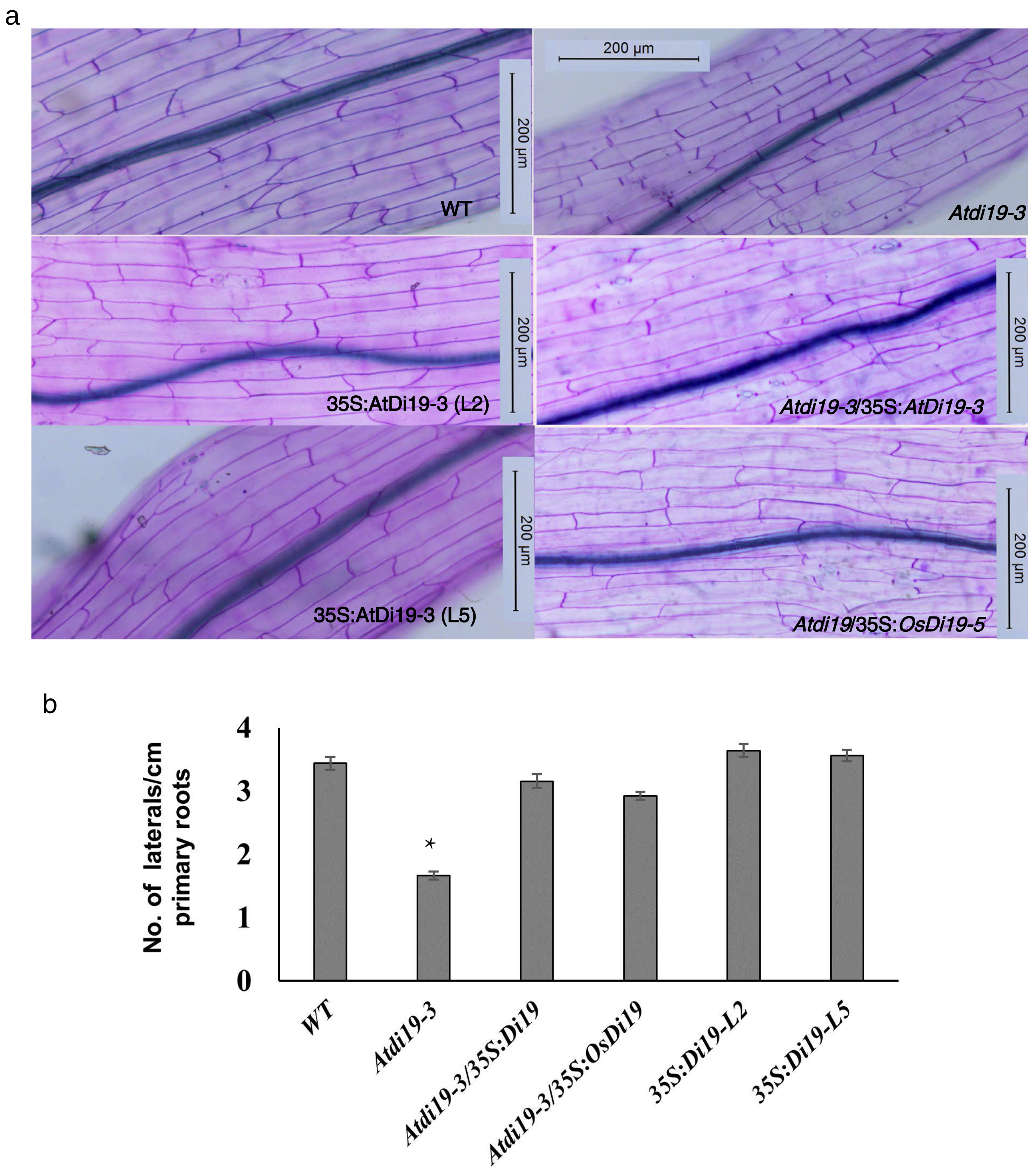

Supplementary Fig S4. **(a)** Enlarged view of the hypocotyl epidermal cells of 7-day-old light grown *Arabidopsis* seedlings. **(b)** Comparison of lateral root density of *Atdi19-3*, overexpression lines *35:Di19* (L2 and L5), complementation lines and wild-type. Mean (±SE) of three independent experiments with 15 seedlings in each was plotted as error bars.

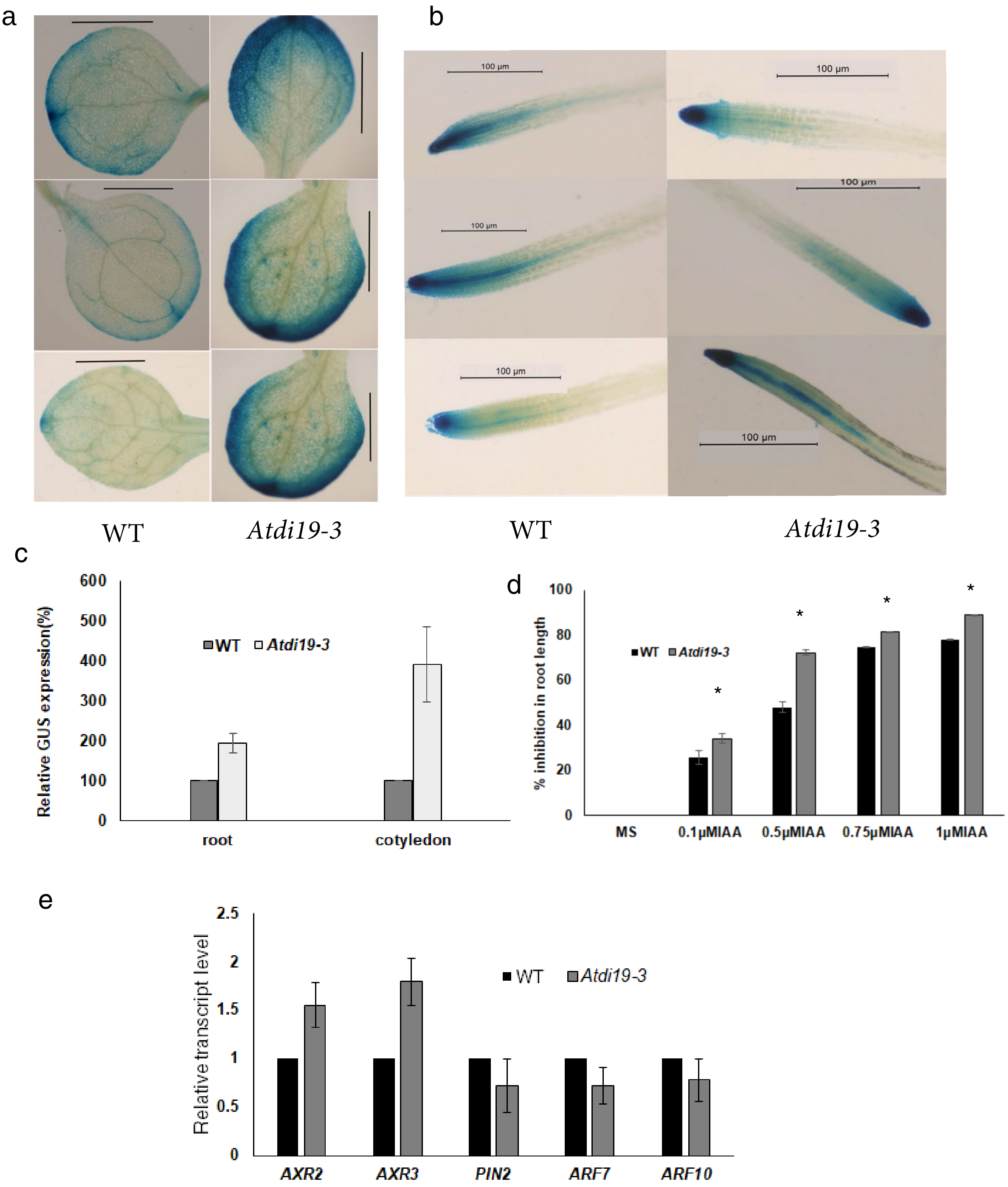

Supplementary Fig S5. Multiple DR5::GUS/Col-0 and DR5::GUS/*Atdi19-3* lines used to examine GUS activity in 10-day-old seedlings. Expression of DR5::GUS reporter activity in **(a)** cotyledon and **(b)** root in F2 generation plants harbouring DR5::GUS in *Atdi19-3* mutant background. Left panel shows GUS induction in DR5::GUS/Col-0 line. **(c)** The intensity of GUS staining in roots and cotyledons of *Atdi19-3* plants was compared to the WT using ImageJ software. **(d)** Root growth inhibition in WT and *Atdi19-3* seedlings under different IAA concentrations. Statistical significance done by student's t-test ($P < 0.05$) (*wild-type vs. mutant). Mean values $\pm$ SE are plotted in both graphs. **(e)** Relative transcript level of *AXR2*, *AXR3*, *PIN2*, *ARF7* and *ARF10* in 10-day-old light grown seedlings of *Atdi19*-3 and wild-type. Mean +SD was plotted as error bars.

–

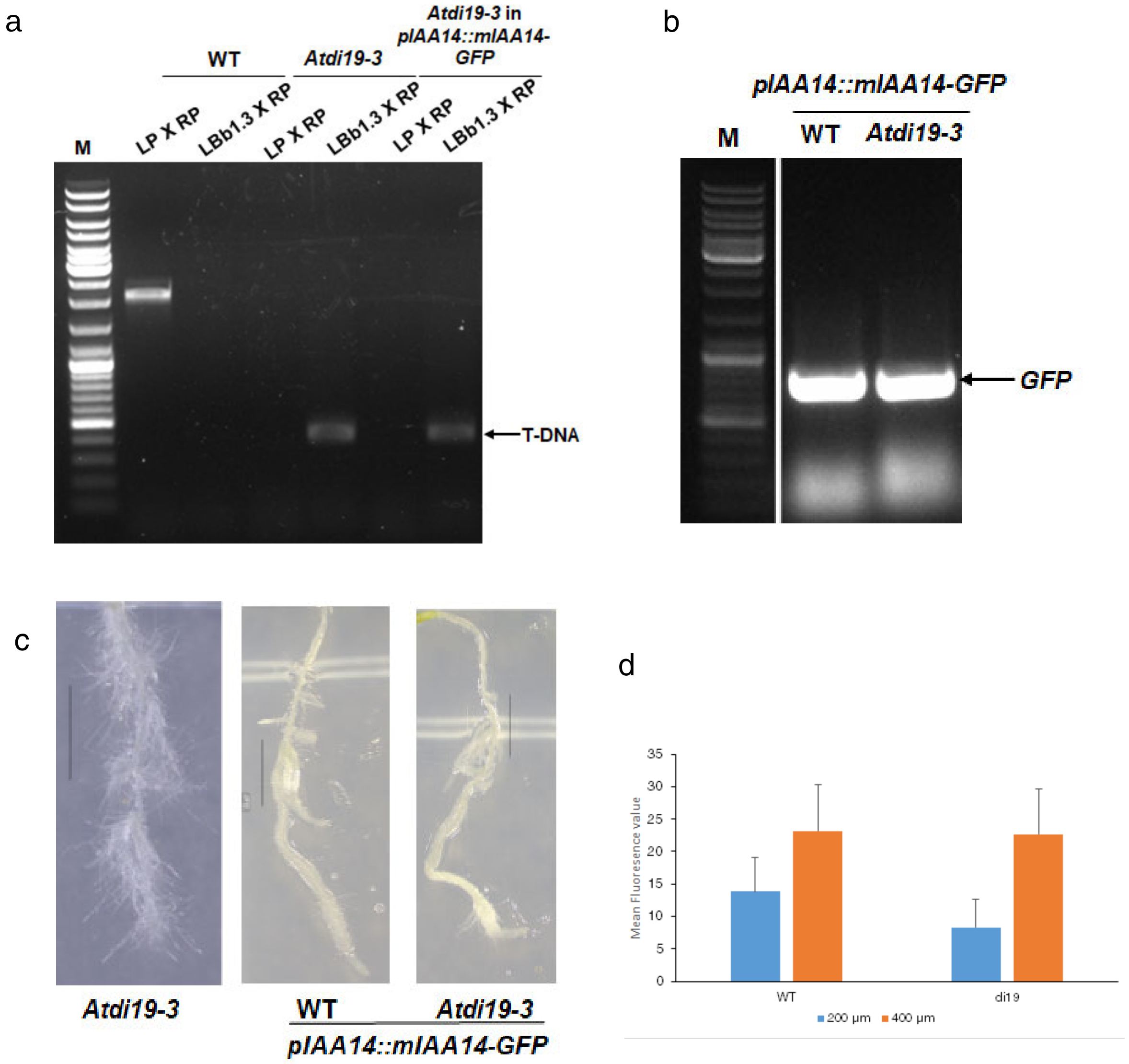

Supplementary Fig S6. **(a)** Picture showing bands corresponding to T-DNA insertion amplified using PCR from genomic DNA in *Atdi19* mutant and *Atdi19* expressing *pIAA14:mIAA14-GFP* . **(b)** Picture showing bands corresponding to *GFP* amplified using PCR from genomic DNA in *pIAA14:mIAA14-GFP* and *Atdi19/pIAA14:mIAA14-GFP*. **(c)** Magnified view of roots from *Atdi19-3* mutant seedlings and WT or *Atdi19-3* mutant expressing *pIAA14::mIAA14-GFP* grown in presence of 1µM NAA. **(d)** Mean fluoresence intensity of GFP measured between root tip till a distance of 200 µm and at a distance of 400 µm from the root tip measured using Leica LAS AF software. The plot shows mean values ± standard deviation.

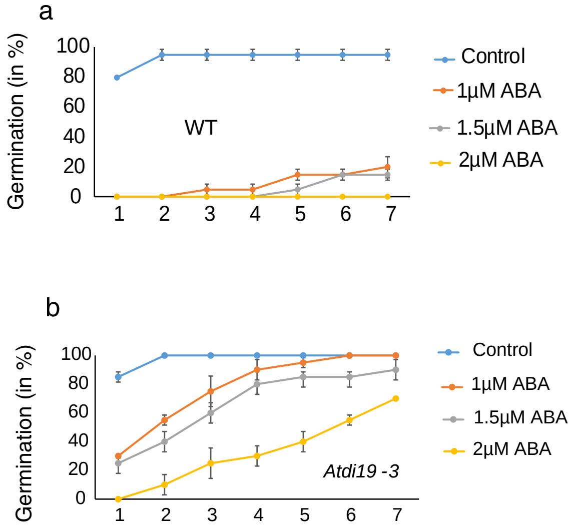

Supplementary Fig S7. Emergence of radical from **(a)** wild-type seeds and **(b)** *Atdi19-3* seeds in presence of different concentration of ABA. Germination was monitored for 7 days. Values plotted are Mean ± SE from 3 different experiments taking 20 seeds each time.

AtIAA14-AtDi19-3

(a)
AtDi19-
nEYFPC1/
AtIAA14-
cEYFPC1

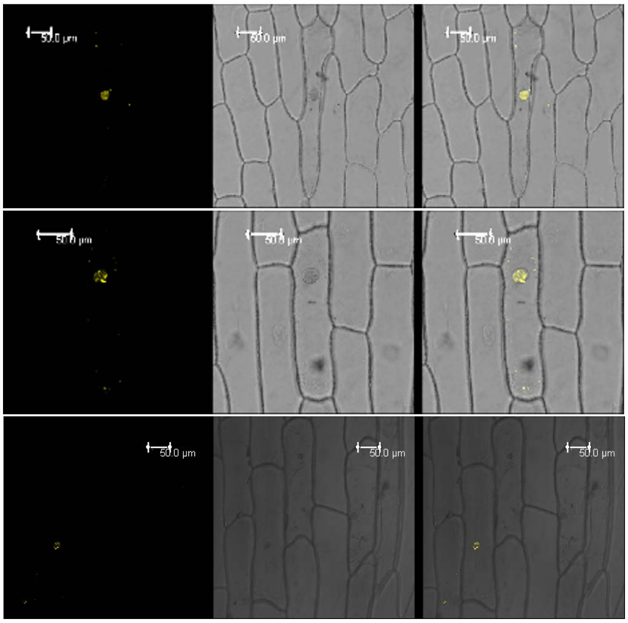

Fluoresence Bright Field Overlay

OsIAA13-OsDi19-5

(b)
IAA13-
cEYFPC1/
OsDi19-
nEYFPC1

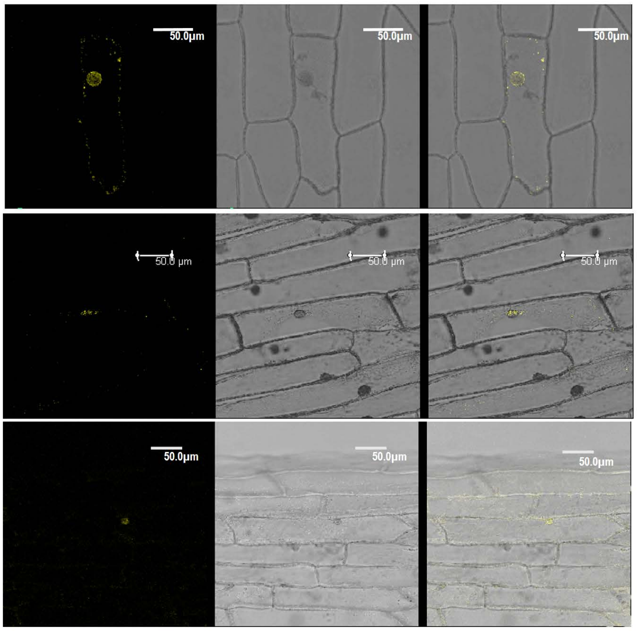

Fluoresence Bright Field Overlay

Supplementary Fig S8. Bimolecular fluorescence complementation analysis of OsIAA13/AtIAA14 and OsDi19/AtDi19. Confocal images of onion epidermal cells bombarded with **(a)**AtIAA14/cEYFPC1 and AtDi19-3/nEYFPC1;**(b)** IAA13/ cEYFPC1 and OsDi19-5/nEYFPC1; Bar is 50µm

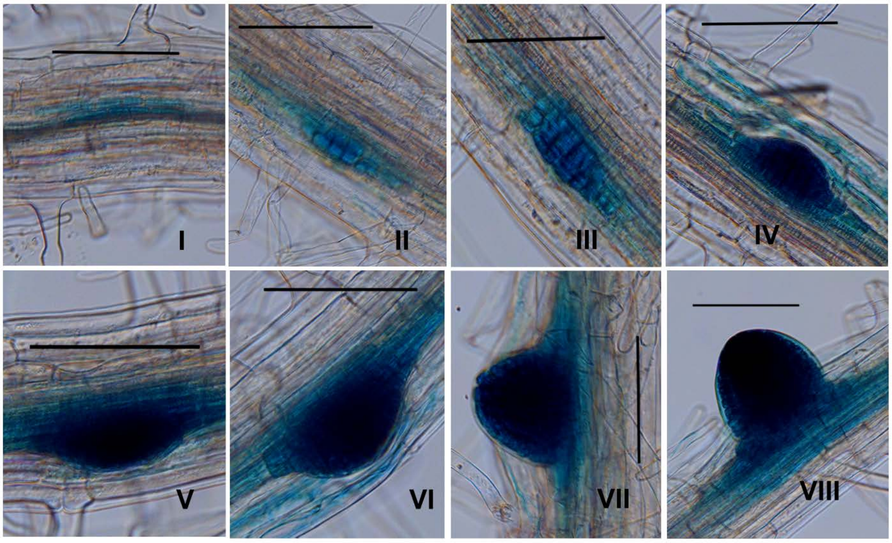

Supplementary Fig S9. Different stages of lateral root primordium emergence in *Arabidopsis.*

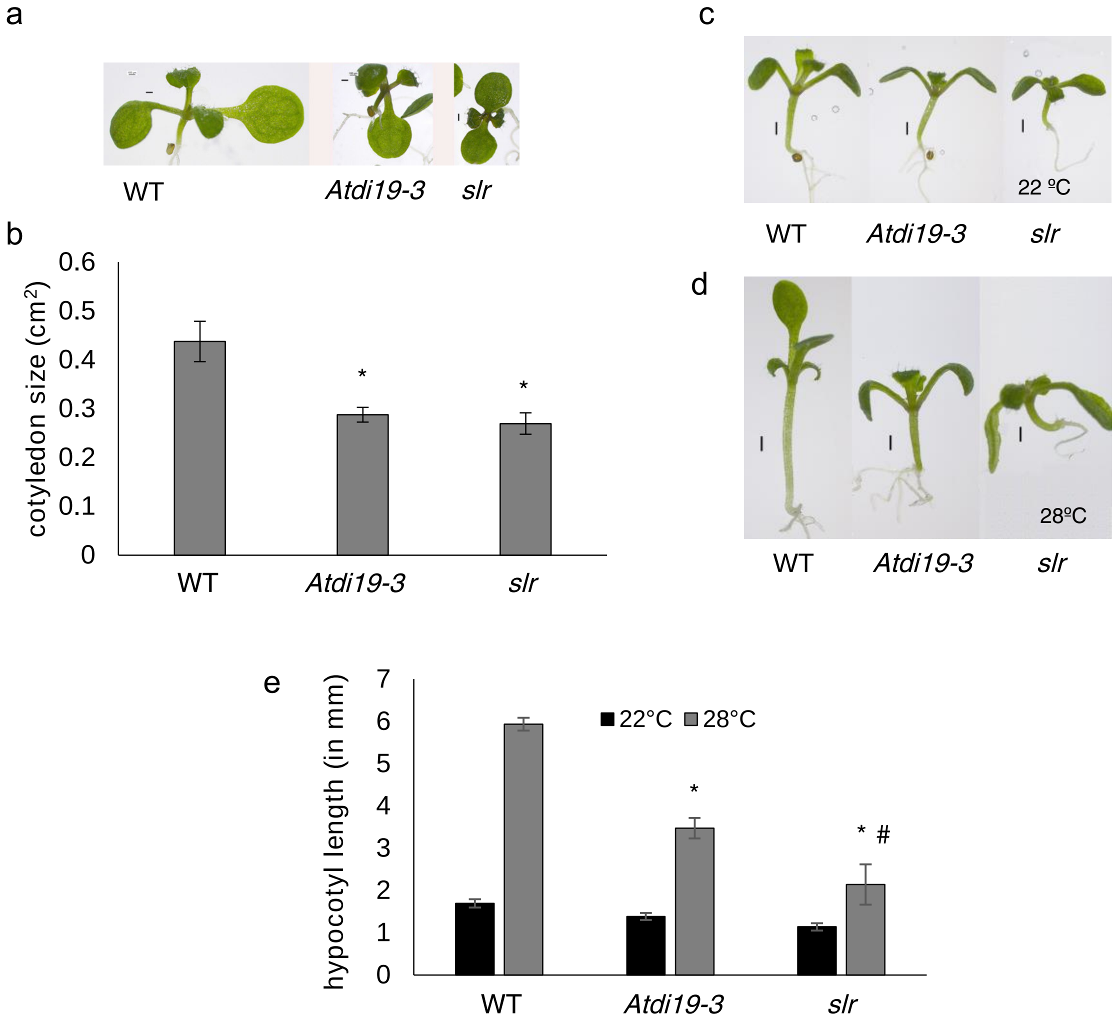

Supplementary Fig S10. Comparative growth analysis of *Arabidopsis slr* mutant and *Atdi19-3* with wild type under light conditions.

(a) Phenotype of 7-day-old seedlings, **(b)** cotyledon area, **(c-e)** hypocotyl elongation of 7-day-old light grown seedlings at 22ºC and 28ºC. Mean with SD of 3 independent experiments with 30 seedlings in each set was plotted. Statistical significance done by student's t-test ($P < 0.05$) (*wild-type vs. mutant), (# *slr* vs. *Atdi19-3*). Mean ± SE of three biological replicates was plotted.

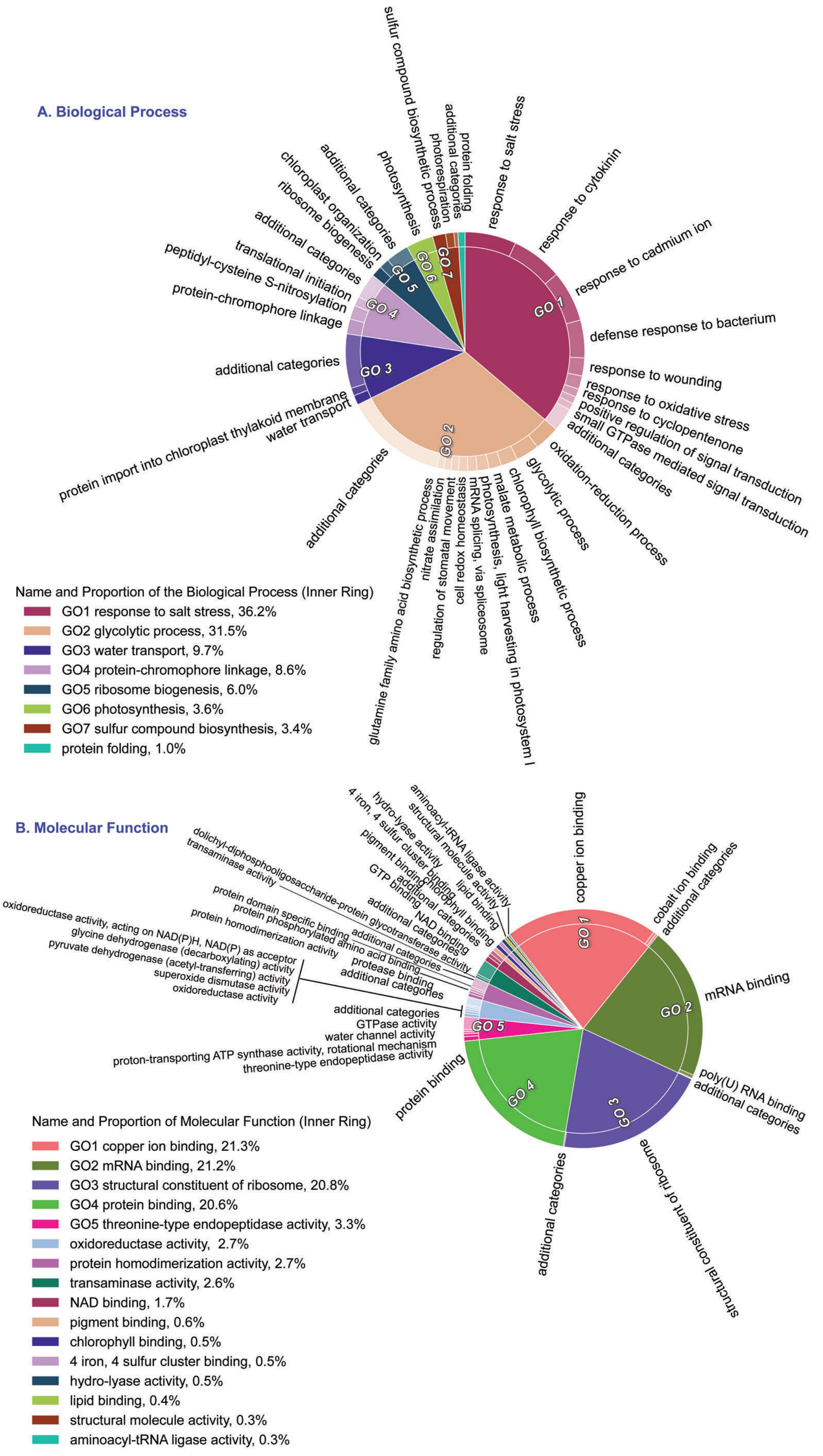

Supplementary Fig S11. GO terms enrichment among DEGs for *Atdi19-3* vs WT under the categories (A) Biological Process and (B) Molecular Function

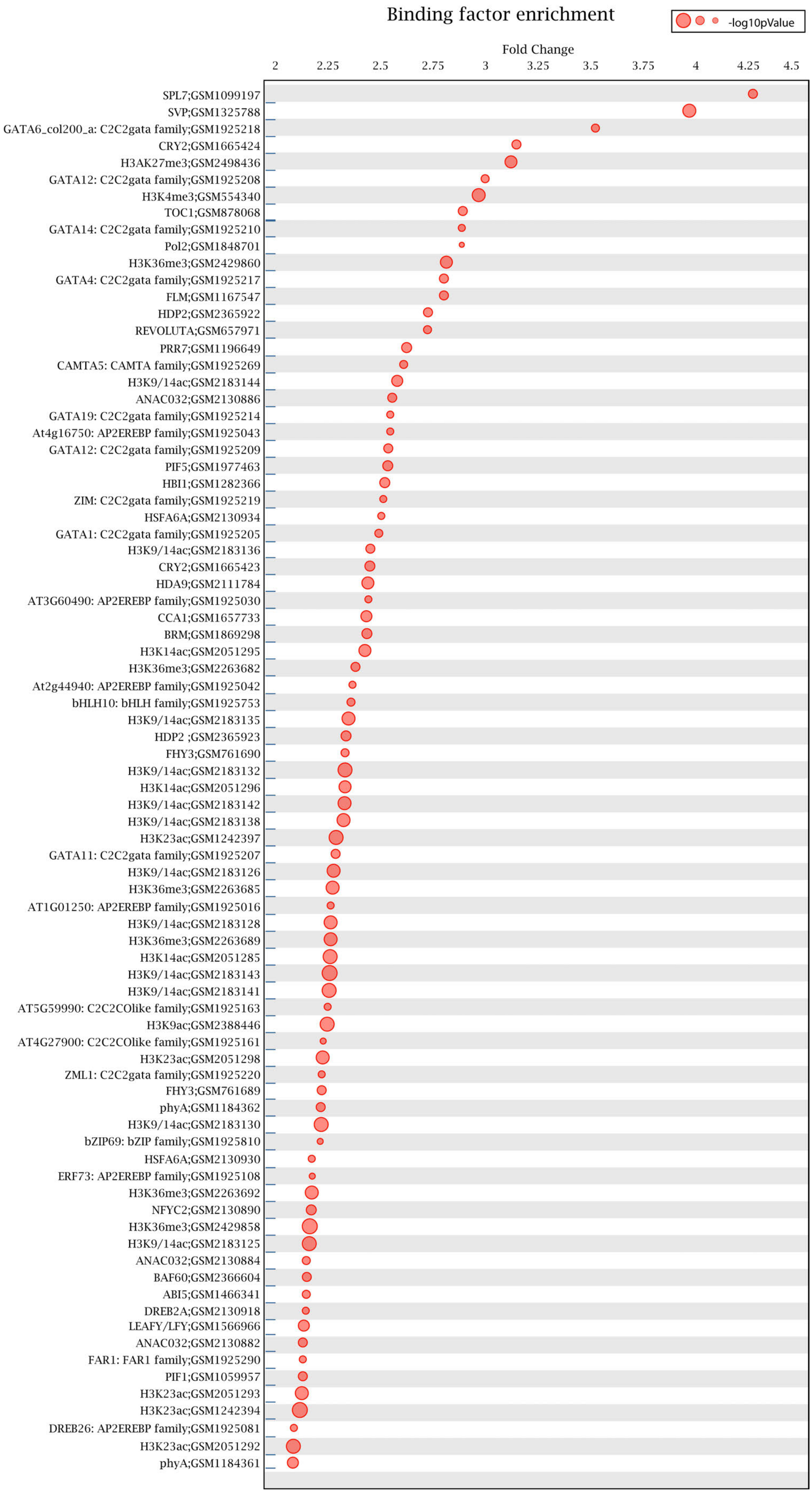

Supplementary Fig S12. Binding factor enrichment analysis among DEGs for *Atdi19-3* vs WT.
